# Development of a red fluorescent light-up probe for highly selective and sensitive detection of vicinal dithiol-containing proteins in living cells†
†Electronic supplementary information (ESI) available: Experimental procedures, characterization data, and additional spectra. See DOI: 10.1039/c5sc02824h



**DOI:** 10.1039/c5sc02824h

**Published:** 2015-10-23

**Authors:** Yuanyuan Wang, Xiao-Feng Yang, Yaogang Zhong, Xueyun Gong, Zheng Li, Hua Li

**Affiliations:** a Key Laboratory of Synthetic and Natural Functional Molecule Chemistry of Ministry of Education , College of Chemistry & Materials Science , Northwest University , Xi'an 710069 , P. R. China . Email: xfyang@nwu.edu.cn; b College of Life Sciences , Northwest University , Xi'an 710069 , P. R. China; c College of Chemistry and Chemical Engineering , Xi'an Shiyou University , Xi'an 710065 , P. R. China . Email: huali@nwu.edu.cn

## Abstract

Vicinal dithiol-containing proteins (VDPs) play a key role in cellular redox homeostasis and are responsible for many diseases. Here, we develop a red fluorescent light-up probe **FAsH** for the highly selective and sensitive detection of VDPs using the environment-sensitive 2-(4-dimethylaminophenyl)-4-(2-carboxyphenyl)-7-diethylamino-1-benzopyrylium (**F1**) as the fluorescent reporter and cyclic dithiaarsane as the targeting unit. **FAsH** is almost nonfluorescent in aqueous solution. However, it exhibits intense fluorescence emission upon binding to reduced bovine serum albumin (rBSA, selected as the model protein). The fluorescence intensity of **FAsH** is directly proportional to the concentration of rBSA over the range of 0.06–0.9 μM, with a detection limit (3*δ*) of 0.015 μM. Importantly, the fast kinetics of binding between **FAsH** and VDPs (∼2.5 min) enables the dynamic tracing of VDPs in biological systems. Preliminary experiments show that **FAsH** can be used for the no-wash imaging of endogenous VDPs in living cells. In addition, our study shows that **F1** presents both high environment-sensitivity and good fluorescence properties, and is promising for the development of no-wash fluorescent light-up probes for target-specific proteins in living cells.

## Abstract 2

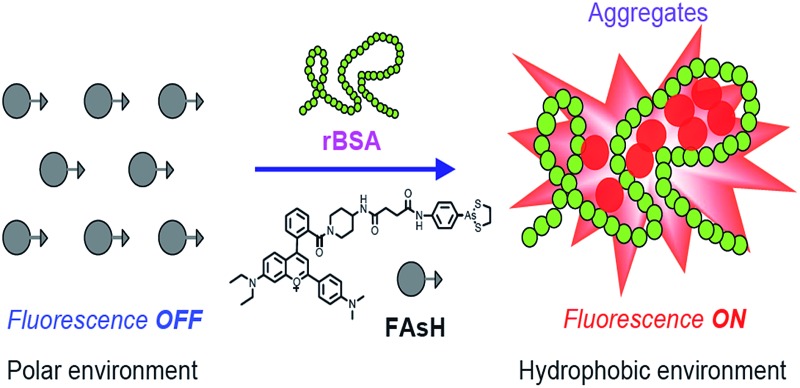
An environment-sensitive red fluorescent light-up probe for vicinal dithiol-containing proteins (VDPs) in living cells has been successfully developed.

## Introduction

Vicinal dithiol-containing proteins (VDPs) are proteins that contain two space-closed cysteine (Cys)-sulfhydryl groups. In general, the vicinal thiols are derived from cysteines that are sequence proximal but may be formed by the juxtaposition of cysteinyl residues – present in distant segments of the same or different polypeptides.^1^ As the reductive end of this redox buffer network, VDPs can undergo reversible oxidative conversion to the intra- or interprotein disulfides and are highly effective in regulating the redox environment of internal cellular compartments under normal conditions.^2^ In addition, VDPs hold a particularly prominent position in protein synthesis and function, and are responsible for many diseases such as cancer,^3^ diabetes,^4^ stroke,^5^ and neurodegeneration.^6^ Therefore, to explore the essential roles of VDPs in cellular redox homeostasis and protein function in living cells, there is a strong desire to develop fluorescent probes for the sensitive and selective sensing of VDPs in living cells.

In recent years, two strategies have been adopted for the development of fluorescent probes for VDPs. One method employs two maleimide groups as the recognition unit, which quenches the probe's fluorescence until they both undergo thiol addition during the labeling reaction. These fluorogenic probes have been used to label vicinal thiol-containing peptides/proteins.^7^ However, intracellular labeling still remains a challenge for these probes since intracellular glutathione (GSH) (1–10 mM) can undergo a similar addition reaction, thus leading to a nonspecific fluorescent labeling reaction.^7*b*^ Alternatively, 1,3,2-dithiarsenolane was incorporated into a variety of fluorophores to develop fluorescent probes for VDPs which have been used to identify VDPs in living cells.^8^ The method employs the fact that trivalent arsenicals can bind to vicinal thiol proteins with high affinities,^9^ whereas proteins with thiols that are not vicinal (referred to as monothiols) interact weakly with arsenicals.^10^ However, a potential drawback of these fluorescent probes is the strong background fluorescence from the unreacted probes inside cells which hinders the identification of labeled proteins.^8a–c^ To circumvent this problem, a tedious washing procedure (>15 min) is required to remove the unbound probes in order to reduce the background fluorescence, which will inevitably delay the acquisition of microscopic data and thus makes the measurements prone to artifacts. To overcome this deficiency, a ratiometric fluorescent probe for VDPs was recently developed by Huang *et al.* based on the fluorescence resonance energy transfer (FRET) mechanism.^8*d*^ Although ratiometric probes can overcome the influence of a variety of factors such as instrumental efficiency, environmental conditions and the probe concentration, the proposed probe exhibits only moderate fluorescence variations (*ca.* 6-fold) upon binding to VDPs. Furthermore, the probe shows a sluggish response to VDPs (>60 min), which makes it unsuitable for the real time monitoring of VDPs inside living cells. Therefore, it is highly desirable to develop fluorescence turn-on probes for intracellular VDPs that have the combination of high selectivity, fast labeling rates, and high fluorescent turn-on ratios.

Recently, a simple strategy for designing fluorescence turn-on probes for selective protein detection has been developed by taking advantage of environment-sensitive fluorophores,^11^ which generally involves the incorporation of an environment-sensitive fluorophore into a ligand specific to the target protein. Typically, these probes exhibit very weak fluorescence in polar and protic environments, while the fluorescence is enhanced when the environment becomes hydrophobic or less polar. They could provide a fluorogenic response to their immediate environment, resulting in a variety of applications in bioanalytical chemistry. This light-up strategy has paved a new way for the detection of targeting proteins with high sensitivity and selectivity.

Although several environment-sensitive fluorophores have been reported,^12^ they show some crucial drawbacks. First, most of those used for light-up probe design are limited to those with blue or green emission, whereas far-red and near-infrared (NIR) dyes are advantageous for cellular studies due to lower photodamage, light scattering, and autofluorescence in living systems.^13^ Second, sensitivity to solvent polarity of these dyes is frequently not enough to detect subtle changes in the environment of the biomolecule of interest. Finally, polarity-sensitive dyes with red emission generally exhibit relatively lower sensitivity to polarity compared to blue dyes.^12*b*^ Therefore, currently intensive research is focusing on the design of environment-sensitive fluorophores with red-shifted emission.

**Scheme 1:**
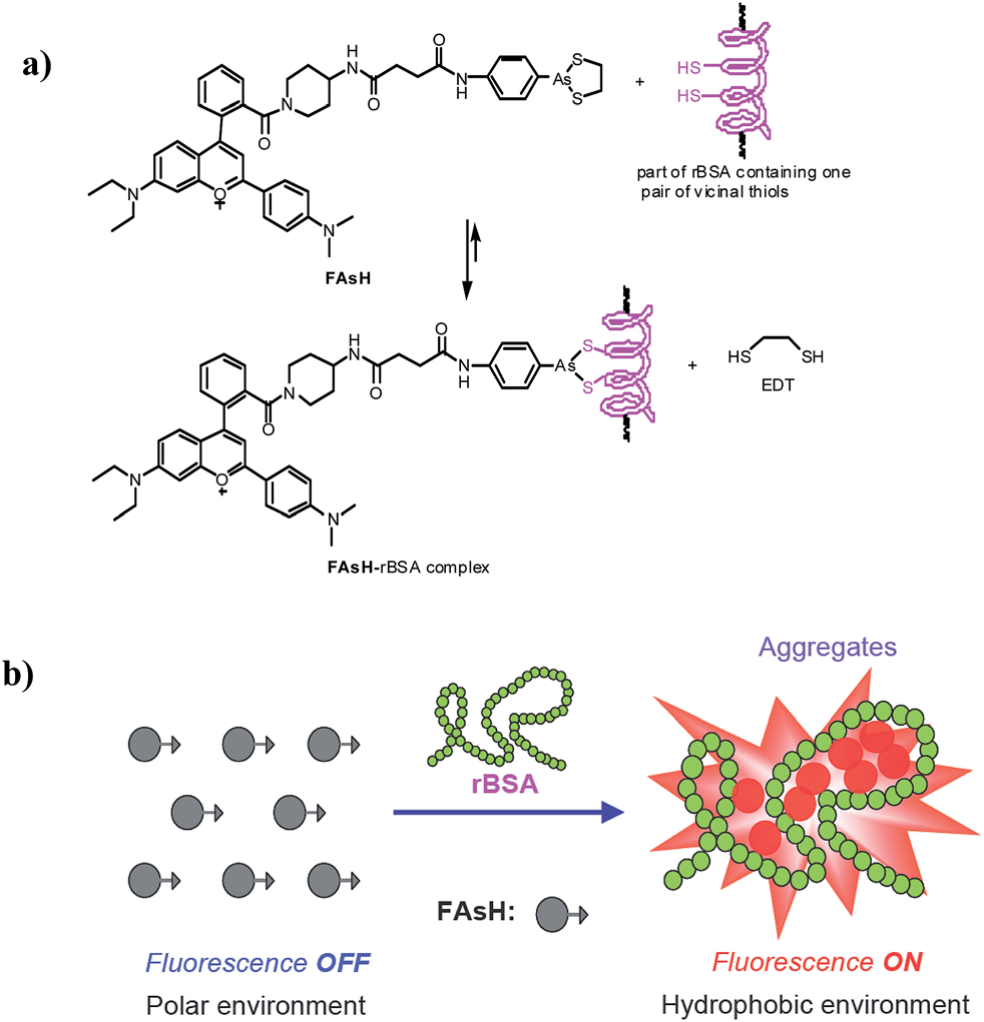
(a) Proposed reaction between **FAsH** and rBSA; (b) schematic illustration of the fluorescence turn-on mechanism for rBSA detection with **FAsH**.

Herein, we report the design and synthesis of a red fluorescent light-up probe for the rapid detection of VDPs both *in vitro* and *in vivo* with excellent sensitivity and specificity. In the proposed sensing system, 2-(4-dimethylaminophenyl)-4-(2-carboxyphenyl)-7-diethylamino-1-benzopyrylium (**F1**) was selected as the environment-sensitive fluorescence reporter and cyclic dithiaarsane as the specific ligand for VDPs. Our rationale is depicted in Scheme 1. We envisioned that the selective binding of protein vicinal dithiols to the trivalent arsenical of **FAsH** would bring the fluorophore into the hydrophobic protein domain, and the hydrophobic environment would cause the fluorophore to emit strong fluorescence. In contrast, in the absence of the target protein, the probe would remain in aqueous solution and should emit only weak fluorescence. Based on the above mechanism, we created a selective fluorescence turn-on probe toward VDPs inside living cells with no-wash procedures. Compared with the reported fluorescent probes, **FAsH** is cell-permeable and shows a rapid response toward VDPs with high sensitivity. Moreover, the proposed probe can operate in the red region, which is favorable for biological applications *in vitro* and *in vivo*. The proposed probe has been used for rapid no-wash imaging of VDPs in living cells.

## Results and discussion

### Probe design

**Fig. 1:**
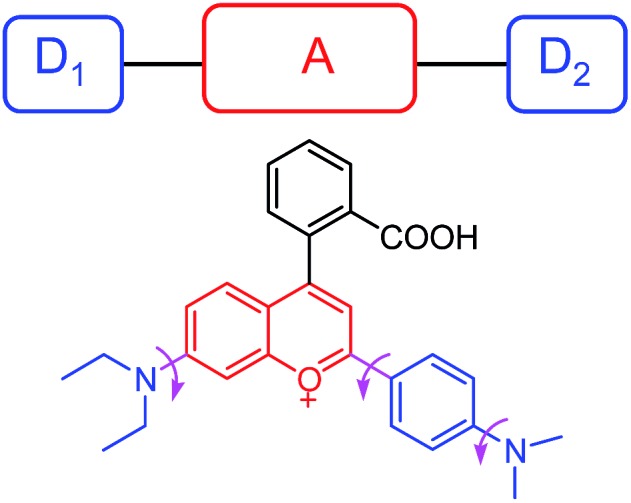
Schematic illustrating the rotational freedom and the electron donor–acceptor–donor (D_1_–A–D_2_) system in **F1**.

In this work, we constructed a novel fluorescent light-up probe for the selective detection of VDPs inside living cells by adopting a fluorogenic mechanism based on an environment-sensitive fluorophore. To obtain an optimal response, there is a strong need to select environment-sensitive dyes presenting both high environment-sensitivity and good fluorescence properties. In this contribution, we focused our interest on a flavylium fluorescent dye **F1**, because it can be regarded as an electron acceptor system (benzopyrylium cation) connected to two different electron donor units (dimethylaminophenyl and diethylamino groups) (Fig. 1), which is a typical characteristic of representative solvatochromic fluorescent molecules.^14^ Although the biological applications of benzopyrylium dyes have been shown recently,^15^ their environment-sensitive behavior remains unexplored.

**Fig. 2:**
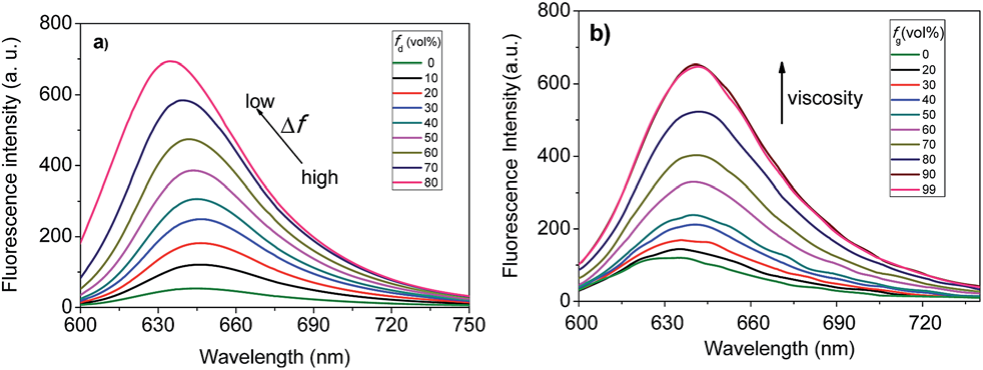
(a) Fluorescence spectra of **F1** in water/1,4-dioxane solvent mixtures with different fractions of 1,4-dioxane (*f*_d_). (b) Fluorescence spectra of **F1** in methanol/glycerol solvent mixtures with different fractions of glycerol (*f*_d_). **F1**, 1.0 μM; *λ*_ex_ = 580 nm.

We then studied the solvatochromic properties of **F1** by measuring its absorption and emission spectra in different proportions of water and 1,4-dioxane with different polarities. As shown in Fig. S1 (ESI†), all the absorption spectra have maxima at about 598 nm, and there are no significant changes observed in the solutions with different polarities. In contrast, solvent polarity had a dramatic effect on the emission spectra of **F1**. When the orientation polarizability (Δ*f*) of the solution decreased from 0.32 (99% water) to 0.292 (20% water),^16^ the maximum emission wavelength of **F1** shifted from 646 to 635 nm, concomitant with a gradual increase in fluorescence intensity (Fig. 2a). The fluorescence intensity of **F1** at 635 nm increased by a factor of 14.2. The above results reveal that **F1** is a polar-sensitive (solvatochromic) fluorescent dye.

Moreover, the multiple electron-donating groups in **F1** are linked to the benzopyrylium unit *via* a single bond. Thus, it features high rotational flexibility and possesses two different twisted intramolecular charge transfer (TICT) channels within the whole molecule (involving twisting of the dimethylaminophenyl and diethylamino groups, respectively, as shown in Fig. 1).^17^ These intramolecular rotations lead to the nonradiative deactivation of the fluorescent excited state, which might be another cause for the fluorescence quenching of **F1** in aqueous solution. To test this assumption, we checked the effect of solvent viscosity on **F1** emission. The intramolecular rotation process is reported to be influenced by the viscosity of the medium: the higher the viscosity of the medium, the slower the intramolecular rotation and hence the stronger the **F1** emission.^18^ We then evaluated the viscosity effect on the emission behavior of **F1** in methanol/glycerol mixtures with different fractions of glycerol (*f*_g_). As shown in Fig. 2b, the emission intensity of **F1** is greatly enhanced as the solvent viscosity increases from 0.60 (methanol) to 950 cP (99% glycerol) at room temperature (25 °C),^19^ which is typically observed for molecular rotors. These experimental results support the fact that **F1** is a viscosity-sensitive dye.

Since **F1** features a dual dependency of emission intensity on both solvent polarity and viscosity, we thus expect that it would hold great promise in the development of environment-sensitive fluorescent probes compared to the traditional solvatochromic fluorescent dyes. Next, we checked the possible nonspecific interactions of **F1** with serum proteins by introducing bovine serum albumin (BSA, 1.0 mg mL^–1^) to the aqueous solution of **F1**. As shown in Fig. S2 (ESI†), only negligible changes in the emission intensity (2.6-fold increase) of **F1** were observed, suggesting there are few nonspecific interactions between **F1** and BSA. This is crucial for the development of probes for *in vivo* imaging, as a high nonspecific background signal is the main reason for their failure. Finally, **F1** contains a carboxylic acid group, which enables facile attachment of the recognition moiety. On the basis of the aforementioned results, **F1** was selected as the fluorophore to construct the probe.

**Scheme 2:**
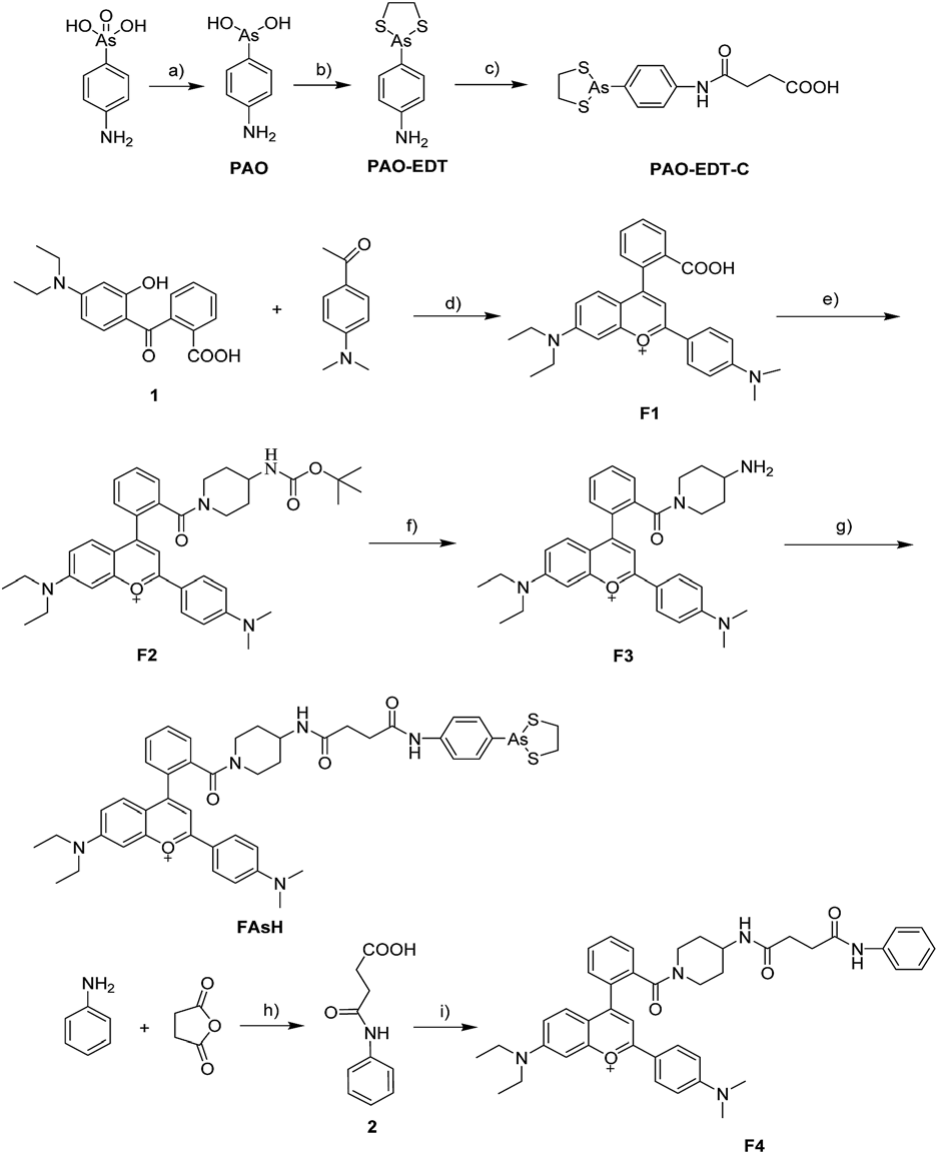
Synthesis of **FAsH** and **F4**. Reagents and conditions: (a) phenylhydrazine, methanol, reflux, 60 min; (b) ethanedithiol, ethanol, reflux, 10 min, 79%; (c) succinic anhydride, toluene, reflux, 3 h, 97%; (d) concentrated H_2_SO_4_, 90 °C, 1.5 h, 67%; (e) 4-(*N*-Boc-amino)piperidine, EDC, HOBt, CH_2_Cl_2_, RT, 6 h, 51%. (f) CF_3_COOH, CH_2_Cl_2_, RT, overnight, 73%; (g) **PAO-EDT-C**, EDC, HOBt, CH_2_Cl_2_, RT, 4 h, 54%; (h) succinic anhydride, toluene, reflux, 3 h, 96%; (i) **F3**, EDC, HOBt, CH_2_Cl_2_, RT, 6 h, 51%. Counterions are omitted for clarity. EDC = 1-ethyl-3-(3-dimethylaminopropyl) carbodiimide hydrochloride, HOBt = 1-hydroxybenzotriazole.

Additionally, we selected 2-(4-aminophenyl)-1,3,2-dithiarsolane (**PAO-EDT**) as the recognition unit because its As(iii) center can selectively discriminate vicinal dithiols from other forms of thiols through the interchange of 1,2-ethanedithiol (EDT) in cyclic dithiaarsanes with vicinal dithiols in proteins.^20^ In addition, its 5-membered dithiarsolane ring is a more stable complex compared with the 6- and 7-membered ones.^21^ In view of the above mentioned results, we rationally designed a red fluorescence turn-on probe **FAsH** for VDPs in living cells. The detailed synthetic procedures and characterization of **FAsH** are shown in Scheme 2. Meanwhile, **F4** which lacks the 5-membered dithiarsolane ring in its structure was also prepared for comparison purposes.

### General spectral properties

With **FAsH** in hand, we studied its spectral properties. The absorption maximum of **FAsH** is located at 612 nm in aqueous solution (*ε*_612 nm_ = 2.68 × 10^4^ M^–1^ cm^–1^), which is red-shifted by about 17 nm in comparison with that of **F1** (Fig. S3a, ESI†). Furthermore, the emission spectra of **FAsH** were recorded in CH_2_Cl_2_ and the results are shown in Fig. S3b (ESI†). In agreement with the red-shift in the absorption spectra, the fluorescence spectrum of **FAsH** displays a 10 nm bathochromic shift when compared to **F1**. The optical spectra of **F4** were almost the same as those of **FAsH** under identical conditions. Furthermore, it was observed that the fluorescence intensity of **FAsH** shows no significant difference to that of **F4** (Fig. S3b, ESI†), indicating that no intramolecular fluorescence quenching was induced by the arsenical moiety, which is apparently different from that seen in bisarsenical dyes.^20,22^

### Optical response

**Fig. 3:**
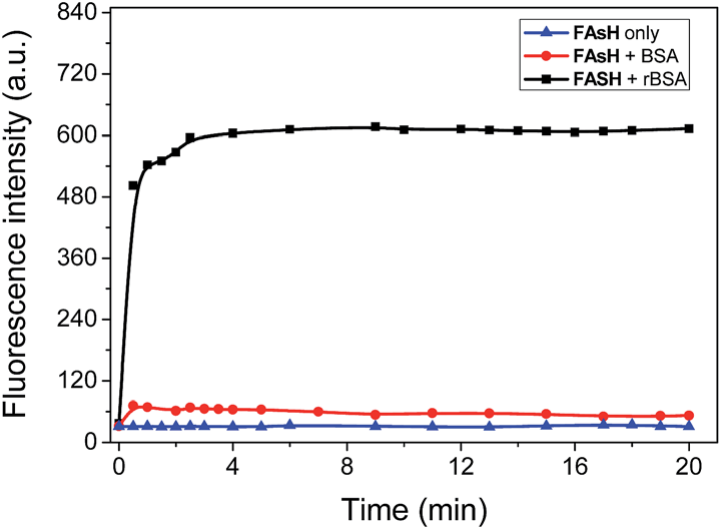
Time course of the fluorescence intensity of **FAsH** (1.0 μM) in the presence of rBSA or BSA (both 0.9 μM) in phosphate buffer (20 mM, pH 7.4, containing 1% acetone as cosolvent). *λ*_ex_/*λ*_em_ = 621/651 nm.

The fluorescence sensing behavior of **FAsH** toward VDPs was examined. Here, reduced bovine serum albumin (rBSA) was selected as the model protein because it has eight vicinal Cys pairs (its structure is shown in Fig. S4, ESI†) after BSA is reduced with tris(2-carboxyethyl)phosphine (TCEP).^7*b*^ Initially, the kinetic behavior of **FAsH** toward rBSA was examined in phosphate buffer (20 mM, pH 7.4, containing 1% acetone as cosolvent). Upon introducing rBSA (1.0 equiv.) to the solution of **FAsH**, a dramatic increase in the fluorescence intensity was observed within 2.5 min, which then reached a plateau as the reaction proceeded, whereas the fluorescence background in the absence of rBSA remained unchanged under identical conditions (Fig. 3). The binding rate of **FAsH** to rBSA is dramatically accelerated in comparison with the previously reported probe^8*d*^ which is very important in monitoring the dynamic changes of VDPs *in situ*. Moreover, upon addition of EDT to the solution of the **FAsH**–rBSA complex, the fluorescence intensity decreased by 42% within 1 min (Fig. S5, ESI†), which proves that the binding of **FAsH** with rBSA is reversible.^20^ By contrast, BSA was added to the solution of **FAsH** and only a negligible fluorescence increase was observed on the time scale of the experiments. These results indicate the high selectivity of **FAsH** toward vicinal dithiols in proteins.

**Fig. 4:**
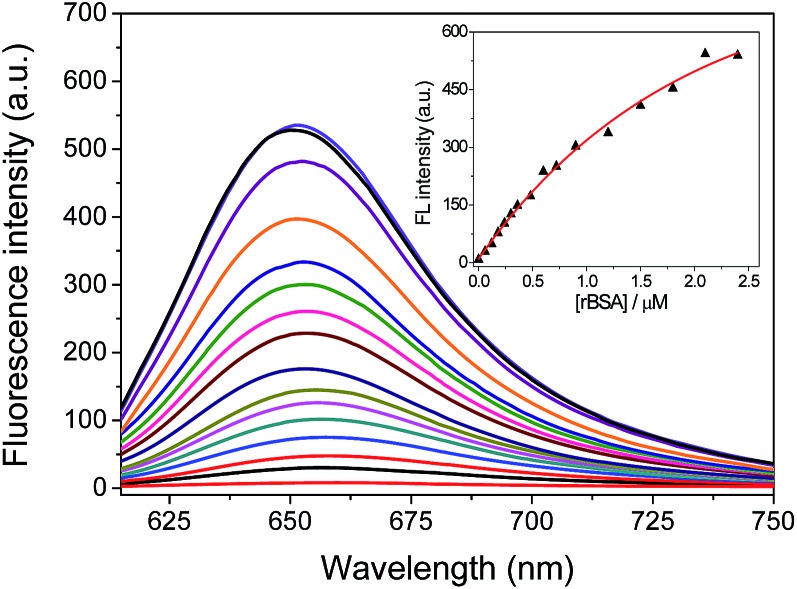
Fluorescence spectra (*λ*_ex_ = 600 nm) changes for **FAsH** (1.0 μM) with the addition of increasing concentrations of rBSA (0–2.4 μM) in phosphate buffer (20 mM, pH 7.4, containing 1% acetone as cosolvent) for 5 min. Inset shows the emission intensity at 651 nm as a function of rBSA concentration.

The fluorescence response of **FAsH** toward rBSA was examined by introducing increasing concentrations of rBSA (0–2.4 μM) to the solution of **FAsH**. As shown in Fig. 4, the free probe gives extremely weak fluorescence in aqueous solution (*φ*_f_ = 0.006, using **F1** in CH_2_Cl_2_ as a reference).^23^ However, the addition of an increasing amount of rBSA to the solution of **FAsH** elicits a gradual increase in the fluorescence intensity and the final enhancement factor is over 70-fold (*φ*_f_ = 0.21). This intensity increase was also accompanied by a hypsochromic shift in the emission spectra from 658 to 651 nm during the titration. The increase in the fluorescence intensity and the hypsochromic shift of the fluorescence emission maxima may be attributed to the binding of **FAsH** to the hydrophobic domain of rBSA. The fluorescence intensity at 651 nm as a function of rBSA concentration was recorded, and a nearly linear relationship over the range of 0.06–0.9 μM was obtained (Fig. S6, ESI†). The detection limit (3*δ*) for rBSA was calculated to be 0.015 μM. These results demonstrate that **FAsH** can detect rBSA with high sensitivity. Furthermore, to test the sensing behavior of **FAsH** toward different VDPs, we examined other reduced forms of proteins (human serum albumin, ovalbumin and lysozome) and found that reduced human serum albumin (rHSA) also induces a dramatic increment in emission intensity, while reduced ovalbumin affords a moderate fluorescence enhancement. In the case of reduced lysozome, a very small increment in emission intensity is observed (Fig. S7, ESI†). This is apparently due to different VDPs having different reactivities with **FAsH**. Thus, **FAsH** is unsuitable for the quantitative determination of VDP content in complicated biological systems because different VDPs will afford different increments in emission intensity.

### Selectivity studies

**Fig. 5:**
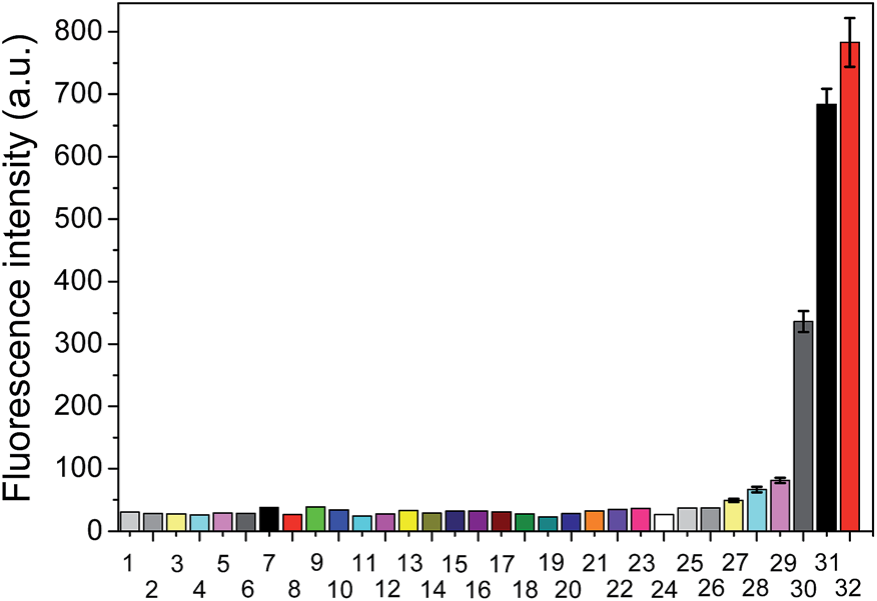
Fluorescence intensity of **FAsH** (1.0 μM) upon mixing with different species in phosphate buffer (20 mM, pH 7.4, containing 1% acetone as cosolvent) for 5 min. 1, blank; 2, Ile; 3, Ser; 4, Arg; 5, Met; 6, Thr; 7, Tyr; 8, Ala; 9, Asp; 10, His; 11, Tpc; 12, Pro; 13, Gly; 14, Glc; 15, Trp; 16, Glu; 17, Lys; 18, Leu; 19, Val; 20, Cys; 21, GSH; 22, Hcy; 23, TCEP; 24, ascorbic acid (100 equiv. of each); 25, cytochrome c; 26, myoglobin; 27, lysozome; 28, BSA; 29, reduced lysozome; 30, reduced ovalbumin; 31, rBSA; 32, rHSA (1 equiv. of each). *λ*_ex_/*λ*_em_ = 600/651 nm.

**Fig. 6:**
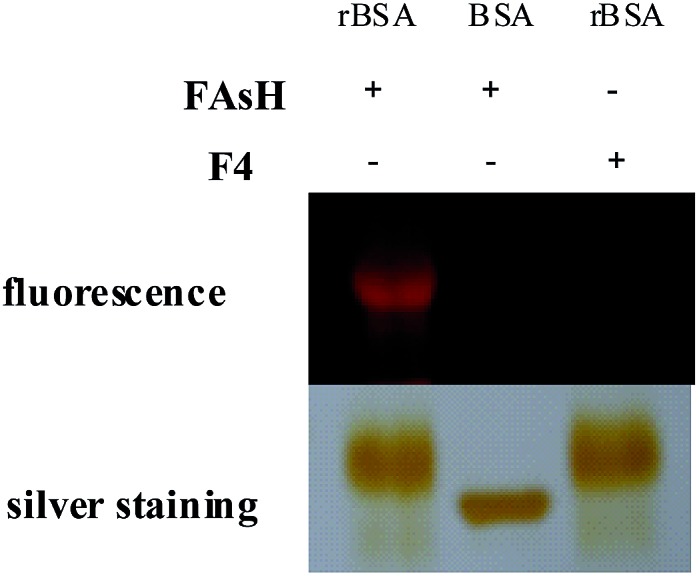
The selective binding of **FAsH** to VDPs was verified by SDS-PAGE. “+”: the compound was present in the detection system; “–”: the compound was absent in the detection system.

To further verify the selectivity of the probe for VDPs, **FAsH** was treated with a series of biologically relevant species in phosphate buffer (20 mM, pH 7.4). As shown in Fig. 5, only VDPs (rBSA, rHSA and reduced ovalbumin, 1 equiv. of each) induce a significant fluorescence increase, while other amino acids (Ile, Ser, Arg, Met, Thr, Tyr, Ala, Asp, His, Tpc, Pro, Gly, Glc, Trp, Glu, Lys, Leu, Val, Cys, Hcy, GSH, 100 equiv. of each), TCEP, ascorbic acid (100 equiv. of each) and proteins (cytochrome c, myoglobin, lysozyme and BSA, 1 equiv. of each) afford no obvious changes in emission intensity. Furthermore, **FAsH** can still retain its sensing behavior toward VDPs even in the presence of a large amount of biothiols (GSH, Cys and Hcy) or reductant (ascorbic acid) (Fig. S8, ESI†), which further confirms the high selectivity of **FAsH** towards VDPs over other cellular thiol-containing compounds. The specificity of **FAsH** toward VDPs was further verified by sodium dodecyl sulfate polyacrylamide gel electrophoresis (SDS-PAGE). As shown in Fig. 6, a fluorescence band was observed in the lane loaded with rBSA and **FAsH**, whereas no visible emission was observed in the case of BSA, which contains only one Cys residue (monothiol protein). Furthermore, no fluorescence band was observed when control compound **F4** was used for rBSA labeling, which provides strong evidence that the 5-membered dithiarsolane ring in **FAsH** is responsible for the specific binding of VDPs. By contrast, these protein bands were observed in the gel after silver staining which proves that the fluorescence band is related to the formation of the rBSA–**FAsH** complex. The excellent selectivity of **FAsH** toward VDPs can be explained by the fact that the cyclic dithioarsinite complexes (**FAsH**–rBSA) formed between trivalent arsenicals and vicinal thiols are markedly more stable than the noncyclic products formed from monothiols due to entropic considerations.^24^

### Mechanistic study

**Fig. 7:**
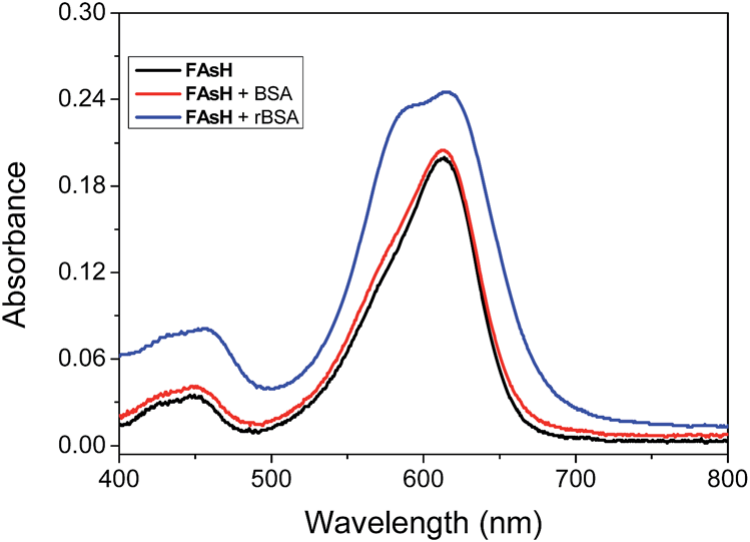
Absorption spectra of **FAsH** (10 μM) in the presence of rBSA or BSA (both 0.3 μM) in phosphate buffer (20 mM, pH 7.4, containing 1% acetone as cosolvent).

Some experiments were carried out to gain further insight into the fluorogenic response of **FAsH** toward VDPs. The absorption spectra of **FAsH** with the introduction of rBSA were recorded, and it was observed that free **FAsH** exhibits two absorption bands at 448 and 612 nm. Upon addition of rBSA, a new blue-shifted absorption band centered at 589 nm emerged, and all the absorption bands were increased with increasing rBSA concentration (Fig. S9, ESI†). The above spectra variation indicates that **FAsH** forms H-aggregates in the presence of rBSA.^25^ Moreover, the aggregates’ formation was evident from the trailing absorption trace of the probe solution in the presence of rBSA due to the light-scattering effects of the dye nanoparticles (Fig. 7 and S9, ESI†). In the case of BSA, almost no spectral changes were observed, suggesting that there is almost no interaction between **FAsH** and BSA. These results indicate that rBSA can induce the aggregation of **FAsH** selectively. The aggregation was further confirmed using dynamic light scattering (DLS) measurements, and the solution of **FAsH** (2.0 μM) with rBSA (1.5 μM) shows an average particle size of 180.3 ± 7.3 nm (Fig. S10, ESI†). The above experimental results support the rBSA-induced aggregation of **FAsH**, which can then restrict the intramolecular rotation and thus lead to a great enhancement in the emission intensity.^26^

Furthermore, to prove the fluorescence enhancement of the sensing process is caused by the hydrophobic pocket of rBSA, guanidine hydrochloride (GdnHCl), a strong protein denaturant, was introduced into the solution of the **FAsH**–rBSA complex, and a significant decrease in emission intensity was observed (Fig. S11, ESI†). This is apparently due to the unfolding of rBSA and the hydrophobic pocket in rBSA is destroyed.^27^ As a result, the probe gets more exposed to the polar environment, which is undesirable for fluorescence emission. The above results reveal the essential role of the hydrophobic cavities of the protein folding structure in the present sensing system. Collectively, we can conclude that the fluorescence enhancement of the present sensing system is achieved by reducing the charge transfer between the fluorophore and the polar media and restricting the intramolecular rotations *via* aggregation simultaneously (Scheme 1b).

### Application of **FAsH** in biological systems

In order to show its application in biological samples, we examined the feasibility of **FAsH** for VDP assays in fetal bovine serum (FBS) samples. The introduction of **FAsH** to FBS solution induced a slight fluorescence enhancement. However, upon adding dithiothreitol (DTT, 5 mM) to the above mixture, the fluorescence intensity increased progressively (Fig. S12, ESI†). As a control, it was observed that DTT alone exhibits no fluorescence increase in **FAsH** solution. The above experiments prove that **FAsH** can selectively respond to VDPs in biological samples.

**Fig. 8:**
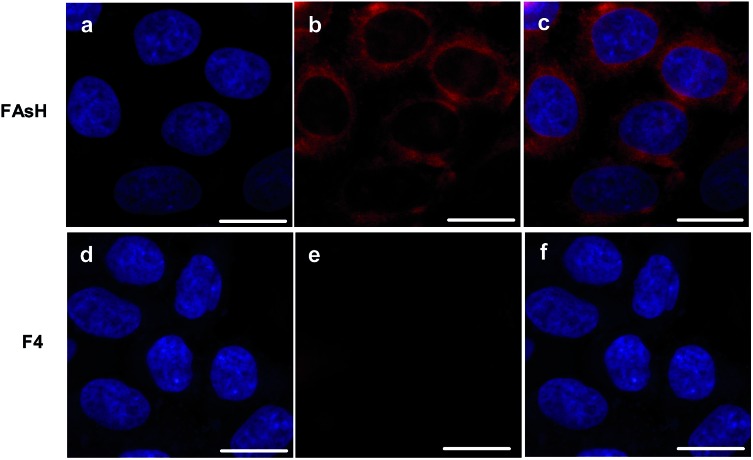
Confocal fluorescence images of intracellular VDPs in SMMC-7721 cells by **FAsH** or **F4**. (a) and (d) The cells were stained with DAPI (1.0 μg mL^–1^); (b) and (e) the cells were stained with **FAsH** and **F4** (both 5.0 μM), respectively; (c) overlay of (a) and (b); (f) overlay of (d) and (e). Scale bar: 15 μm.

Next, some experiments were performed to evaluate **FAsH** in live-cell imaging assays using human hepatoma cells (SMMC-7721) as a model cell line. Initially, the cytotoxicity of **FAsH** was evaluated using a standard MTT assay. Although **PAO** is quite toxic, the results showed that **FAsH** has minimal cytotoxicity at concentrations of 2–20 μM (Fig. S13, ESI†). This is apparently due to the EDT caging unit, which prevents **FAsH** from exerting acutely toxic effects.^20^ Next, SMMC-7721 cells were incubated with **FAsH** for 20 min in PBS, and a strong fluorescence signal was observed. Furthermore, in a control experiment, the cells were pretreated with 30 μM **PAO** (a selective VDP binding reagent) to reduce the amount of intracellular free VDPs prior to incubation with **FAsH**. A pronounced fluorescence quenching was observed (Fig. S14, ESI†), which reveals that the above fluorescence emission (Fig. 8) is indeed induced by VDPs. By contrast, **F4** was used for cell staining and it affords negligible fluorescence emission under the same conditions (Fig. 8). The semiquantitative calculation of the averaged fluorescence intensity was further conducted. The emission intensity of **FAsH**-stained cells is about 19-fold higher than that of **F4**-treated cells (Fig. S15, ESI†). The significant difference in emission intensity indicates the selective binding of **FAsH** to endogenous VDPs inside live cells, and this selective binding is apparently due to the 5-membered dithiarsolane ring. These results demonstrate the capacity of **FAsH** for *in situ* imaging of VDPs in living cells.

**Fig. 9:**
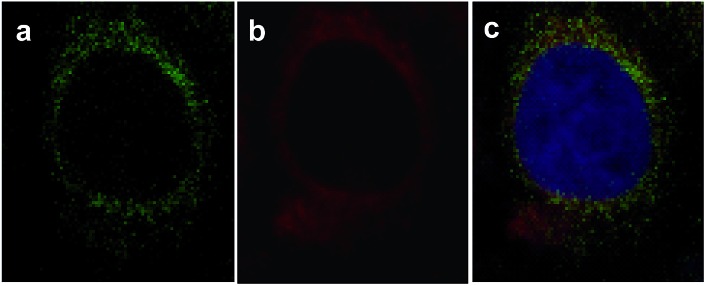
**FAsH** colocalizes to the mitochondria in SMMC-7721 cells. The cells were stained with (a) 2.5 μg mL^–1^ rhodamine 123, (b) 5.0 μM **FAsH**, and (c) overlay of (a) and (b) with DAPI (1.0 μg mL^–1^) stain.

To further investigate the subcellular localization of VDPs, a commercially available mitochondrial tracker (rhodamine 123) was used for a colocalization study with confocal microscopy. As displayed in Fig. 9, the observed fluorescence signal from **FAsH** extensively overlaps with that of rhodamine 123, implying that **FAsH**-labeled VDPs are mainly localized to the mitochondria of these live cells. Furthermore, nuclear staining with DAPI indicates that the cells are viable throughout the imaging experiments. The above experiments prove that there is an abundance of VDPs distributed in the mitochondria of SMMC-7721 cells, which is consistent with previous studies.^8a,28^

## Conclusions

In summary, we have developed a fluorescent light-up probe **FAsH** for selective detection of VDPs using a unique environment-sensitive flavylium dye **F1** as the fluorescent reporter. The probe is almost non-fluorescent in its free form, but exhibits strong fluorescence emission upon specifically binding to VDPs. Therefore, its application no longer requires tedious washing steps during protein labeling, which is favorable for the direct, noninvasive tracing of VDPs in a cellular redox environment. Compared with the widely used solvatochromic fluorescent probes, **FAsH** possesses the characteristics of two types of environment-sensitive fluorophores (molecule rotors and solvatochromic fluorescent dyes) simultaneously. Thus, it shows a more sensitive light-up fluorescence response toward VDPs. Preliminary imaging experiments reveal that **FAsH** can serve as a unique probe for the no-wash visualization of endogenous VDPs in living cells. In addition, **FAsH** has the advantage of rapid binding kinetics, a high signal-to-noise ratio, red emission and will be an attractive tool for the *in situ* investigation of the essential role of VDPs in intracellular redox homeostasis and the exploration of its diverse pathophysiology.
