# Supplementary material for: Development of a red fluorescent light-up probe for highly selective and sensitive detection of vicinal dithiol-containing proteins in living cells
Source: Chem Sci. 2015 Oct 23;7(1):518–24. doi: 10.1039/c5sc02824h (PMC5519953; doi:10.1039/c5sc02824h)
Supplement: Supplementary file 1 [file SC-007-C5SC02824H-s001.pdf]

Electronic Supplementary Material (ESI) for Chemical Science.
This journal is © The Royal Society of Chemistry 2015

Electronic Supplementary Information

Development of a red fluorescent light-up probe for highly selective and sensitive detection of vicinal dithiol-containing proteins in living cells

Yuanyuan Wang, Xiao-Feng Yang,* Yaogang Zhong, Xueyun Gong, Zheng Li, Hua Li*

[a] Key Laboratory of Synthetic and Natural Functional Molecule Chemistry of Ministry of Education, College of Chemistry & Materials Science, Northwest University, Xi'an 710069, P.R. China. xfyang@nwu.edu.cn (X. F. Yang).

[b] College of Life Sciences, Northwest University, Xi'an 710069, P.R. China.

[c] College of Chemistry and Chemical Engineering, Xi'an Shiyou University, Xi'an 710065, P.R. China.

CONTENTS:

Materials and instruments

General procedure for the spectral measurements

SDS-PAGE and fluorescence imaging of gels

Cell Cultures and fluorescence imaging

MTT assay

Colocalization of **FAsH** and rhodamine 123

Determination of the fluorescence quantum yield of **FAsH** and **FAsH**-rBSA complex

Synthesis of **FAsH** and **F4**

References

Figure S1. Absorption spectra of **F1** in water/1, 4-dioxane solvent mixtures.

Figure S2. Fluorescence spectra of **F1** in the absence and presence of BSA.

Figure S3. Absorption and fluorescence spectra of **F1**, **F4** and **FAsH**.

Figure S4. Vicinal cysteine pairs in rBSA.

Figure S5. Fluorescence time profile of **FAsH** upon binding to and dissociating from rBSA.

Figure S6. The linear relationship between the fluorescence intensity and rBSA concentration.

Figure S7. Fluorescence response of **FAsH** to different proteins and their reduced forms.

Figure S8. Fluorescence response of **FAsH** to rBSA in the presence of different competing analytes.

Figure S9. Absorption spectra of **FAsH** in the presence of different concentrations of rBSA.

Figure S10. DLS analysis of the particle size distribution of **FAsH** in the presence of rBSA.

Figure S11. Fluorescence spectra of **FAsH** with the addition of rBSA and subsequently treating with GndHCl.

Figure S12. Time course of the fluorescence intensity of **FAsH** in the presence of FBS, DTT or the mixture of FBS and DTT.

Figure S13. Cell viability estimated by MTT assay.

Figure S14. Confocal fluorescence images of intracellular VDPs in SMMC-7721 cells by **FAsH**.

Figure S15. Semiquantitative determination of endogenous VDPs in SMMC-7721 cells.

Figure S16 – S28. NMR and MS data for compound **PAO-EDT**, **F1**, **F2**, **F3**, **FAsH**, compound **2** and **F4**.

Materials and instruments

Unless stated otherwise, all the chemicals were obtained from commercial suppliers and used without further purification. Rhodamine 123 and DAPI were obtained from Sigma-Aldrich. Solvents were dried by standard procedures before use. Other chemical reagents were of analytical grade and commercially available. Double distilled water was used throughout the experiments. **FAsH** and **F4** were dissolved in acetone as stock solution. rBSA (0.15 mM) was prepared and stored according to the literature [S1].

The fluorescence spectra and relative fluorescence intensity were measured with a Perkin Elmer LS-55 fluorescence spectrometer equipped with a xenon lamp excitation source and a Hamamatsu (Japan) 928 PMT. Absorption spectra were recorded using a Shimadzu UV-2550 spectrophotometer. High-resolution mass spectra were collected using a Bruker micrOTOF-Q II mass spectrometer (Bruker Daltonics Corp., USA) in electrospray ionization (ESI) mode. ^{1}H NMR and ^{13}C NMR spectra were recorded at 400 and 100 MHz on an INOVA-400 spectrometer (Varian Unity) respectively, using tetramethylsilane (TMS) as the internal standard. Fluorescence imaging was performed by an Olympus FV1000 confocal laser scanning microscope (Japan). Dynamic light scattering (DLS) measurements were performed using Particle Size & Zeta Potential Analyzer（ZetaPlus, Brookhaven）. The scattering angle was 90°, and the laser wavelength was 660 nm. The pH measurements were carried out on a Sartorius PB-10 pH meter. All the measurements were operated at room temperature (25 °C).

General procedure for the spectral measurements

In a set of test tubes containing 0.5 mL phosphate buffer (0.2 M, pH 7.4) and 50 μL of **FAsH** (0.1 mM), different concentrations of analytes were added and the reaction mixture was diluted to 5.0 mL with H_2O. The resulting solution was well-mixed and kept at room temperature for 5 min, and then the absorption or fluorescence spectra were recorded.

SDS-PAGE and fluorescence imaging of gels

The selectivity of **FAsH** was proved by SDS-PAGE. Samples were labeled in PBS buffer with a final concentration of protein at 3.75 μM, **FAsH** (or **F4**) at 50 μM for 30 min. After labeling, the samples were mixed with 5 × loading buffer, and run on a 10% polyacrylamide resolving gel and a 3% stacking gel. Molecular mass standards (Thermo Scientific, Waltham, USA) were run with all gels. For fluorescence assay, the gel was scanned on the red fluorescence channel (635 nm excitation/650 nm LP emission) using a phosphorimager (Storm 840, Molecular DynamicsInc. USA). After that, the same gel was stained with alkaline silver for comparison.

Cell Cultures and fluorescence imaging

SMMC-7721 cells were seeded in 6-well culture plates containing sterile coverslips and were cultured in RPMI 1640 medium supplemented with 10% (v/v) fetal bovine serum (FBS), penicillin (100 U mL^{-1}) and streptomycin (100 μg mL^{-1}) at 37 ^{o}C in a humidity atmosphere under 5% CO_2 for 24 h. The medium was removed and the adherent cells were washed with PBS buffer (pH 7.4) three times. After the cells were incubated with 5.0 μM **FAsH** (or 5.0 μM **F4**) in serum-free RPMI medium at 37 °C for 20 min, the medium was removed. The cells were further incubated with nucleus staining dye DAPI (1.0 μg mL^{-1}) in the same medium for 10 min. After that, the staining solution was replaced with fresh PBS to remove the remaining free dye. Cell imaging was then performed by an Olympus FV1000 confocal laser scanning microscope immediately. Excitation wavelength for **FAsH**: 635 nm; Emission collection: 655-755 nm; Excitation wavelength for DAPI: 405 nm; Emission collection: 425-475 nm.

MTT assay

The cytotoxicity of **FAsH** was performed by a standard MTT assay. SMMC-7721 cells were seeded in 96-well flat bottom microtiter plates for 24 h before detection with about 80% intensity. The cells were then incubated with various concentrations of **FAsH** (0 – 20 μM) (n = 4) in FBS-free RPMI 1640 medium in a humidified environment containing 5% CO_2 at 37 ^{o}C for 12 h. 20 μL of MTT solution (5.0 mg

mL^{-1}) was then added to each well. After 4 h, 100 μL of the supernatant was removed, and 150 μL of DMSO was added to each well to dissolve the formazan crystals. After that, the plate was shaken for 10 min and then the absorbance was measured at 490 nm with a microplate reader (WD-2102A Microplate Reader, Beijing Liuyi Instruments Inc.).

Colocalization of FAsH and rhodamine 123

For colocalization experiments, SMMC-7721 cells were incubated with 5.0 μM **FAsH**, 2.5 μg mL^{-1} rhodamine 123 in FBS-free RPMI 1640 medium for 20 min. After the above medium was removed, the cells were further incubated with nucleus staining dye DAPI (1.0 μg mL^{-1}) in RPMI 1640 medium for 10 min. Coverslips were then transferred to fresh PBS buffer for imaging. Excitation wavelength for **FAsH**: 635 nm; Emission collection: 655-755 nm; Excitation wavelength for rhodamine 123: 488 nm; Emission collection: 500-600 nm. ImageJ software was used for analysis of the images.

Determination of the fluorescence quantum yield of FAsH and FAsH-rBSA complex

Fluorescence quantum yields of **FAsH** and **FAsH**-rBSA complex were determined by using **F1** (φ_F = 0.76 in CH_2Cl_2) as fluorescence standard [S2]. The fluorescence quantum yields of **F1** and **FAsH**-rBSA were determined in phosphate buffer (pH 7.4, 20 mM, containing 1% acetone as cosolvent). Spectral data of **FAsH**-rBSA complex was measured 10 min after the mixture of 2.4 μM of rBSA with **FAsH** in phosphate buffer. The quantum yield was calculated using the following equation:

$$\varphi_{F(X)} = \varphi_{F(S)}(A_S F_X / A_X F_S)(n_X/n_S)^2$$

Where φ_F is the fluorescence quantum yield, A is the absorbance at the excitation wavelength, F is the area under the corrected emission curve, and n is the refractive index of the solvent used. Subscript "S" and "X" refer to the standard and the unknown compound, respectively.

Synthesis of FAsH and F4

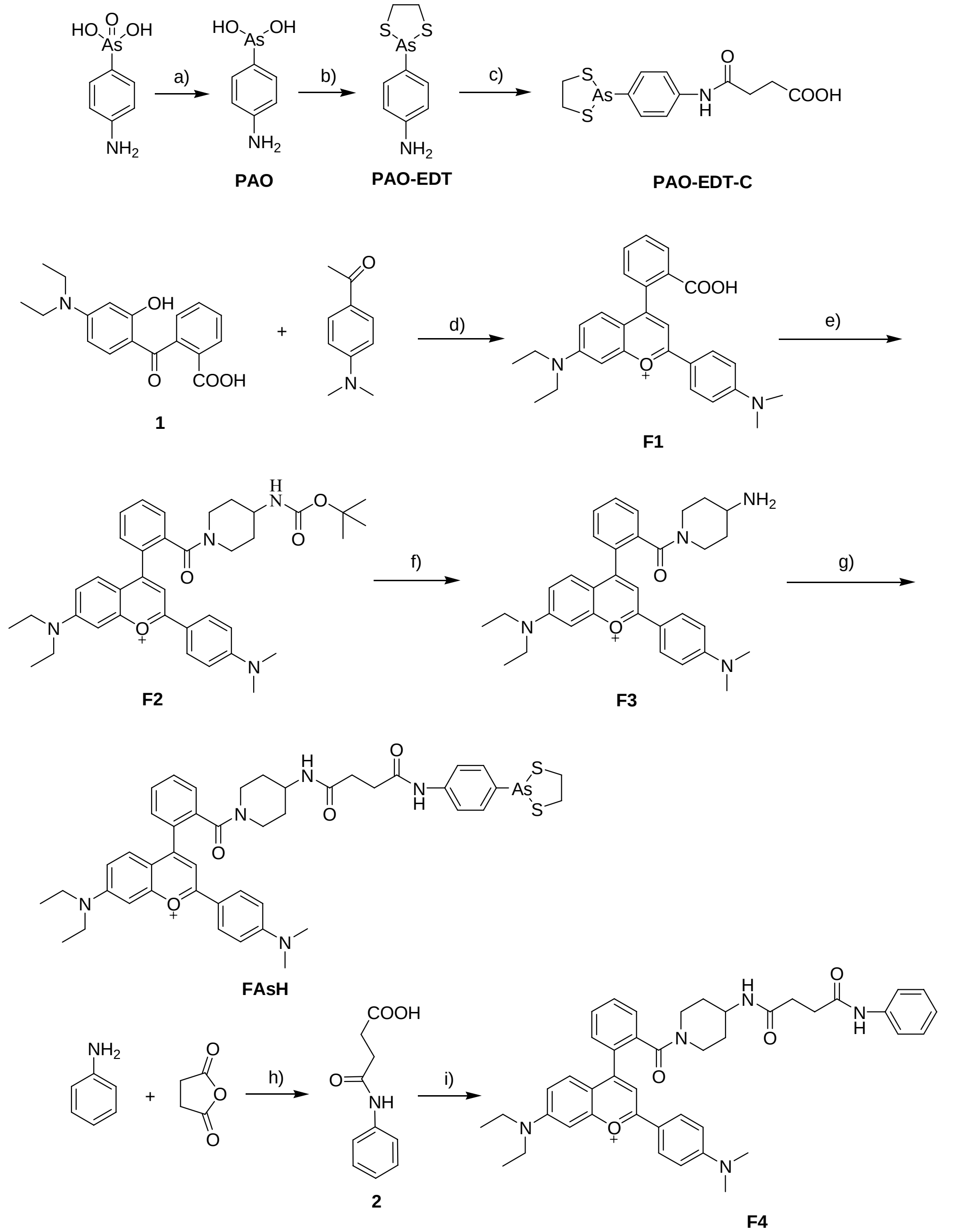

Scheme S1. Synthesis of **FAsH** and **F4**. Reagents and conditions: (a) phenylhydrazine, methanol, reflux, 60 min; (b) ethanedithiol, ethanol, reflux, 10 min, 79%; (c) succinic anhydride, toluene, reflux, 3 h, 97%; (d) concentrated H_2SO_4, 90 °C, 1.5 h, 67%; (e) 4-(N-Boc-amino)piperidine, EDC, HOBt, CH_2Cl_2, RT, 6 h, 51%. (f) CF_3COOH, CH_2Cl_2, RT, overnight, 73%; (g) **PAO-EDT-C**, EDC, HOBt, CH_2Cl_2, RT, 4 h, 54%; (h) succinic anhydride, toluene, reflux, 3 h, 96%; (i) **F3**, EDC, HOBt, CH_2Cl_2, RT, 6 h, 51%. Counterions are omitted for clarity. EDC = 1-ethyl-3-(3-dimethylaminopropyl) carbodiimide hydrochloride, HOBt = 1-hydroxybenzotriazole.

Sythesis of 2- p-aminophenyl-1, 3, 2-Dithiarsenolane (PAO-EDT) [S3]

4-Aminophenylarsenoxide **(PAO)** was prepared according to the literature procedure [S3]. **PAO** (0.96 g, 4.78 mmol) was dissolved in anhydrous ethanol (10 mL) and heated to reflux. Then, ethanedithiol (0.48 mL, 6.30 mmol) was added dropwise to the above solution, and the mixture was refluxed with stirring for 10 min. The reaction mixture was then cooled with ice-cold water, and the resulting precipitate was filtered off to give the crude product, which was further recrystallized from ethanol to afford **PAO-EDT** as a white solid (0.98 g, 79% yield). ^{1}H NMR (400 MHz, DMSO-d_6), δ (ppm): 7.26 (d, J = 7.6 Hz, 2H), 6.55 (d, J = 7.2 Hz, 2H), 5.42 (s, 2H), 3.32-3.27 (m, 2H), 3.26-3.19 (m, 2H). HRMS (ESI): m/z calcd for $C_9H_{10}AsNS_2$ [M + H]$^+$ 259.9549; found: 259.9547.

Sythesis of PAO-EDT-C

PAO-EDT-C was prepared according to the literature procedure [S4]. To a solution of **PAO-EDT** (0.10 g, 0.39 mmol) in toluene (8 mL) was added succinic anhydride (43 mg, 0.42 mmol). The above suspension was reflux for 3 h. After cooled to room temperature, the resulting precipitate was filtered off and dried under vacuum to give **PAO-EDT** as a white solid (0.14 g, 97% yield), which was then used for next step reaction without further purification.

Sythesis of 2-(4-dimethylaminophenyl)-4-(2-carboxyphenyl)-7-diethylamino-1-benzopyrylium (**F1**) [S2]

Compound **F1** was prepared according to the literature procedure [S3]. A mixture of 1-(4-(dimethylamino) phenyl)ethanone (82 mg, 0.5 mmol) and 2-(4-diethylamino-2-hydroxybenzoyl) benzoic acid (**1**) [S5] (0.16 g, 0.5 mmol) in concentrated H_2SO_4 (10 mL) was stirred at 90 °C for 1.5 h under argon atmosphere. After cooling to room temperature, the mixture was then poured in ice-cold water solution (60 mL), and then perchloric acid (70%, 0.5 mL) was added. The resulting precipitate was filtered, washed with cold water, and then dried under vacuum to give **F1** as a purple-red solid (0.18 g, 67% yield), which can be used in the next step

reaction without further purification. HRMS (ESI): *m/z* calcd for $C_{28}H_{29}N_2O_3^+$[M]$^+$ 441.2178; found: 441.2211.

Synthesis of F2

To a mixture of **F1** (90 mg, 0.17 mmol), 4-(N-Boc-amino)-piperidine (50 mg, 0.25 mmol), N-(3-dimethylaminopropyl)-N'-ethylcarbodiimide hydrochloride (EDC, 58 mg, 0.3 mmol), and HOBt (48 mg, 0.3mmol) was added dry CH_2Cl_2 (5 mL). The resulting solution was allowed to stir at room temperature (RT) for 6 h. The reaction solution was then washed with water, dried over anhydrous Na_2SO_4, filtered, and evaporated under reduced pressure. The crude reside was purified by silica gel flash column chromatography (CH_2Cl_2/MeOH = 20:1, v/v) to give compound **F2** (63 mg, 51% yield). ^{1}HNMR (400 MHz, DMSO-d$_6$), δ (ppm): 8.24 (d, *J* = 9.2 Hz, 2H), 7.71-7.69 (m, 3H), 7.60 (br, 1H), 7.56 (d, *J* = 4 Hz, 1H), 7.33-7.30 (m, 2H), 7.21 (d, *J* = 9.2 Hz, 1H), 6.95 (d, *J* = 8.8 Hz, 2H), 6.85 (d, *J* = 6.4 Hz, 1H), 4.05-3.97 (m, 1H), 3.40 (2H), 3.64 (q, *J* = 7.2 Hz, 4H), 3.19 (s, 6H), 1.65 (br, 2H), 1.35(s, 9H), 1.22 (t, *J* = 6.8 Hz, 6H), 1.09 (m, 2H). HRMS (ESI): *m/z* calcd for $C_{38}H_{47}N_4O_4^+$ [M]$^+$ 623.3597; found: 623.3605.

Synthesis of compound F3

Compound **F2** (0.16 g, 0.22 mmol) was dissolved in CH_2Cl_2 (8 mL), and then CF_3COOH (8 mL) was added. The mixture solution was stirred at room temperature overnight. The solvent was then evaporated under reduced pressure, and the resulting reside was purified by silica gel flash column chromatography ($CHCl_3$/MeOH = 10:1, v/v) to give **F3** in 73% yield (0.10 g). ^{1}HNMR (400 MHz, DMSO-d$_6$), δ (ppm): 8.24 (d, *J* = 8.8 Hz, 2H), 8.02 (br, 2H, -NH_2), 7.75-7.71 (m, 3H), 7.60-7.53 (m, 2H), 7.35-7.31 (m, 2H), 7.21 (d, *J* = 8.8 Hz, 1H), 6.95 (d, *J* = 9.2 Hz, 2H), 4.19 (br, 1H) 3.63 (q, *J* = 6.0 Hz, 4H), 3.19 (s, 6H), 1.85 (br, 2H), 1.38 (m, 2H), 1.22(t, *J* = 7.2 Hz, 6H). HRMS (ESI): *m/z* calcd for $C_{33}H_{39}N_4O_2^+$ [M]$^+$ 523.3073; found: 523.3063. (Some peaks of piperidine ring are overlapped with DMSO and H_2O in solvent, so it can not be precisely integrated.)

Synthesis of FAsH

Compound **PAO-EDT-C** (54mg, 0.15 mmol), **F3** (61mg, 0.10mmol), EDC (50 mg, 0.26 mmol) and HOBt (42 mg, 0.26 mmol) were dissolved in dry CH_2Cl_2 (10 mL), and the reaction mixture was allowed to stir at room temperature for 4 h. The reaction solution was then washed with water, dried over anhydrous Na_2SO_4, filtered, and evaporated under reduced pressure. The resulting residue was purified by silica gel flash chromatography (CH_2Cl_2/MeOH = 20: 1, v/v) to afford **FAsH** as a dark blue solid (52 mg, 54% yield**)**. ^{1}HNMR (400 MHz, DMSO-d_6), δ (ppm):10.03 (s, 1H), 8.25 (d, *J* = 8.4 Hz, 2H), 7.84 (d, *J* = 7.2 Hz, 1H), 7.73-7.70 (m, 3H), 7.60-7.53 (m, 5H), 7.34-7.31 (m, 2H), 7.24-7.19 (m, 1H), 7.06 (d, *J* = 9.6 Hz, 1H), 6.96 (d, *J* = 8.4 Hz, 2H), 4.04-3.92 (m, 1H), 3.64 (q, *J* = 7.2, 4H), 3.38-3.35 (m, 2H), 3.19 (s, 6H), 3.17-3.11 (m, 2H), 2.35 (t, *J* = 7.0 Hz, 2H), 1.68 (br, 2H), 1.22(t, *J* = 7.2 Hz, 6H), 1.12 (2H). ^{13}C NMR (100 MHz, DMSO-d_6), δ(ppm): 170.69, 170.58, 166.74, 156.11, 154.93, 154.14, 140.24, 136.87, 131.33, 130.23, 129.57, 129.17, 127.20, 119.64, 118.62, 116.69, 114.74, 112.68, 109.03, 96.53, 46.59, 45.05, 41.36, 31.60, 30.85, 30.16, 12.48. HRMS (ESI): *m/z* calcd for $C_{45}H_{51}AsN_5O_4S_2^+$ $[M]^+$ 864.2598; found: 864.2617.

Synthesis of compound 2

Compound **2** was prepared by the reaction of phenylamine with succinic anhydride using the same procedure described for the synthesis of **PAO-EDT-C**. Yield, 96%. ^{1}H NMR (400 MHz, DMSO-d_6), δ (ppm): 12.13 (s, 1H), 9.94 (s, 1H), 7.57 (d, *J* = 7.6 Hz, 2H), 7.27 (t, *J* = 7.8 Hz, 2H), 7.00 (t, *J* = 7.2 Hz, 1H), 2.55-2.51 (m, 4H).

Synthesis of compound F4

Compound **F4** was prepared via the reaction of **F3** with **F2** using the procedure described for the synthesis of **FAsH.** Yield: 51% ^{1}HNMR (400 MHz, DMSO-d_6), δ (ppm):9.92 (s, 1H), 8.25 (d, *J* = 8.4 Hz, 2H), 7.85 (d, *J* = 7.2 Hz, 1H), 7.73-7.68 (m, 3H), 7.61-7.55 (m, 4H), 7.34-7.22 (m, 5H), 7.07-6.95 (m, 3H), 4.05-3.94 (m, 1H), 3.64 (q, *J* = 6.8 Hz, 4H), 3.19 (s, 6H), 2.37 (t, *J* = 6.8 Hz, 2H), 1.68 (br, 2H), 1.23 (t, *J*

= 6.8 Hz, 6H). HRMS (ESI): *m/z* calcd for $C_{43}H_{48}N_5O_4^+$ $[M]^+$ 698.3706; found: 698.3727.

References

[S1] X. Pan, Z. Liang, J. Li, S. Wang, F. Kong, K. Xu and B. Tang, *Chem. Eur. J.*, 2015, **21**, 2117–2122.

[S2] P. Czerney, G. Graneβ, E. Birckner, F. Vollmer and W. Rettig, *J. Photochem. Photobiol., A,* 1995, **89**, 31-36.

[S3] N. Wimmer, J. A. Robinson, N. Gopisetty-Venkatta, S. J. Roberts-Thomson, G. R. Monteith and I. Toth, *Med. Chem.*, 2006, **2**, 79-87.

[S4] C. Huang, Q. Yin, J. Meng, W. Zhu, Y. Yang, X. Qian and Y. Xu, *Chem. Eur. J.*, 2013, **19**, 7739–7747.

[S5] E. Nakata, Y. Yukimachi, H. Kariyazono, S. Im, C. Abe, Y. Uto, H. Maezawa, T. Hashimoto, Y. Okamoto and H. Hori, *Bioorg. Med. Chem.*, 2009, **17**, 6952-6958.

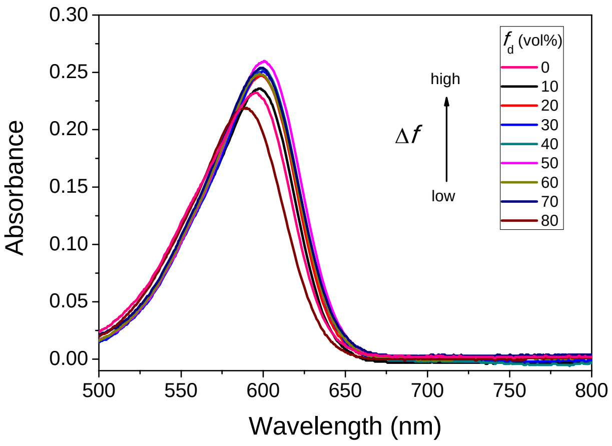

Figure S1. Absorption spectra of **F1** (10 μM) in water/1, 4-dioxane solvent mixtures with different fractions of 1, 4-dioxane (f_d).

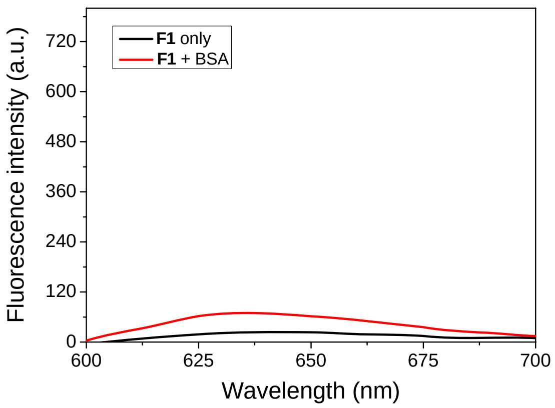

Figure S2. Fluorescence spectra (λ_{ex} =580 nm) of **F1** (1.0 μM) in the absence and presence of BSA (1.0 mg mL^{-1}) in phosphate buffer (20 mM, pH 7.4, containing 1% acetone as cosolvent).

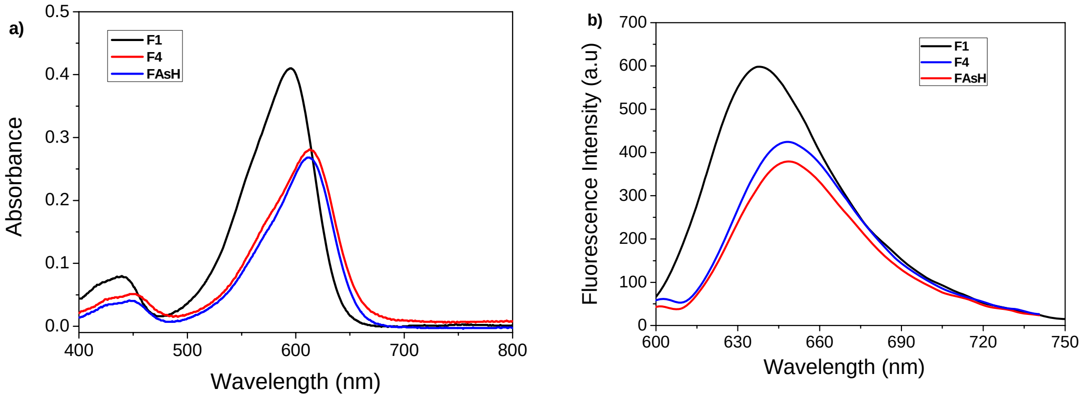

Figure S3. (a) Absorption spectra of **F1**, **F4** and **FAsH** (both 10 μM) in phosphate buffer (20 mM, pH 7.4, containing 1% acetone as cosolvent). (b) Fluorescence spectra (λ_{ex} =580 nm) of **F1**, **F4** and **FAsH** (both 1.0 μM) in CH_2Cl_2.

(1) 53-62; (2) 75-91; (3) 90-101; (4) 123-168; (5) 167-176; (6) 199-245; (7) 244-252; (8) 264-278; (9) 277-288; (10) 315-360; (11) 359-368; (12) 391-437; (13) 436-447; (14) 460-476; (15) 475-486; (16) 513-558; (17) 557-566.

Figure S4. Vicinal cysteine pairs in rBSA. In BSA the disulfide bonds are located in the above positions, and the vicinal cysteine pairs are highlighted yellow.

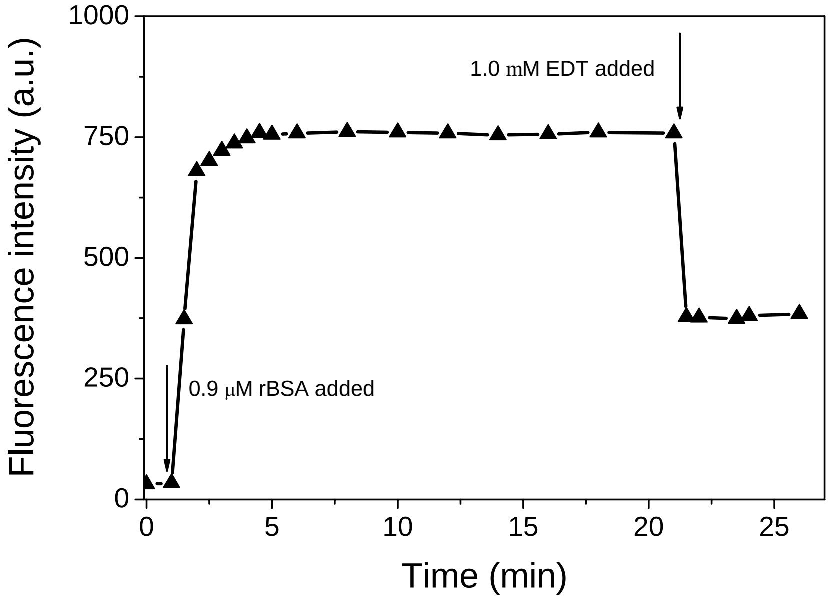

Figure S5. Fluorescence time profile of **FAsH** (1.0 μM) upon binding to and dissociating from rBSA (0.9 μM). The binding was reversed with the addition of EDT (1.0 mM). λ_{ex} / λ_{em} = 621/651 nm.

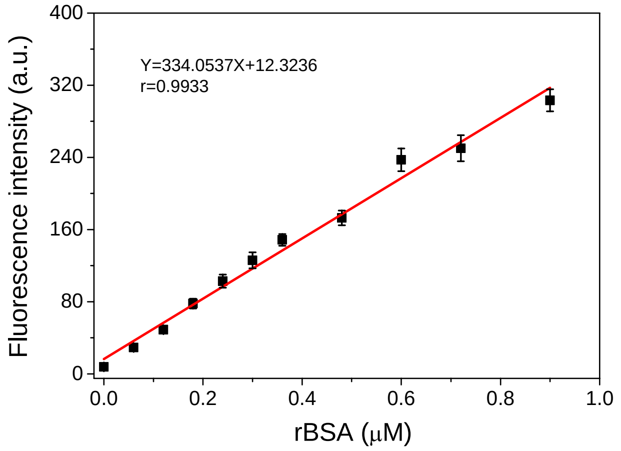

Figure S6. The linear relationship between the fluorescence intensity and rBSA concentration. λ_{ex} / λ_{em} = 621 / 651 nm.

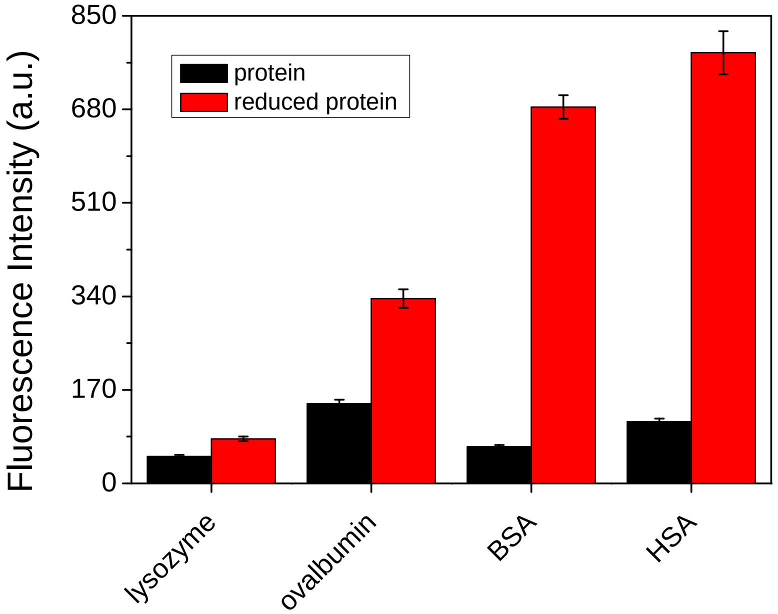

Figure S7. Fluorescence response of **FAsH** (1.0 μM) to different proteins and their reduced forms (1 equiv of each) in phosphate buffer (20 mM, pH 7.4, containing 1% acetone as cosolvent) for 5 min. λ_{ex} / λ_{em} = 600/651 nm. The reduced proteins were prepared by using the same procedure for preparing rBSA.

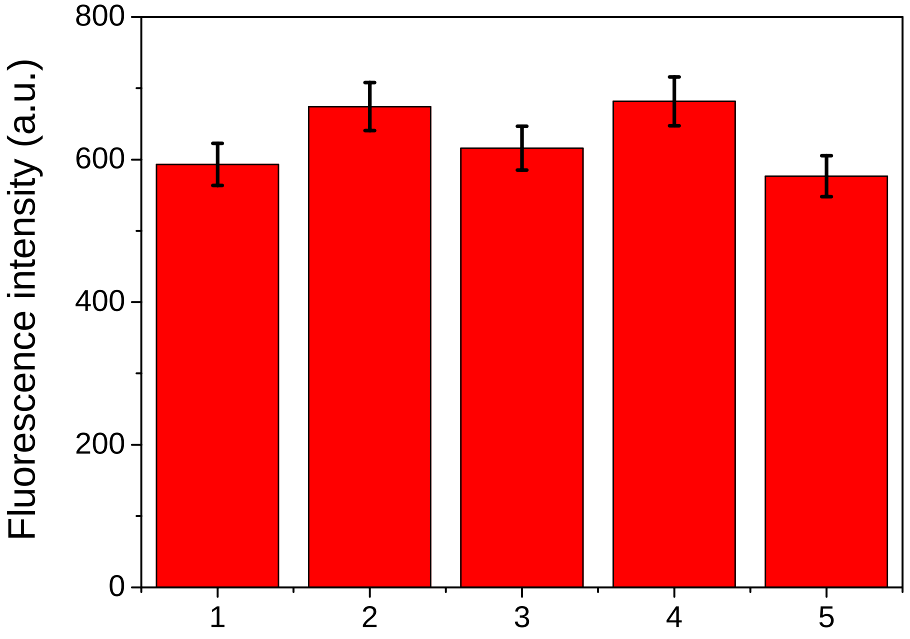

Figure S8. Fluorescence response of **FAsH** (1.0 μM) to rBSA (0.9 μM) in the presence of 1.0 mM of different competing analytes. (1) Free; (2) Cys; (3) GSH; (4) Hcy; (5) ascorbic acid. λ_{ex} / λ_{em} = 600/651 nm.

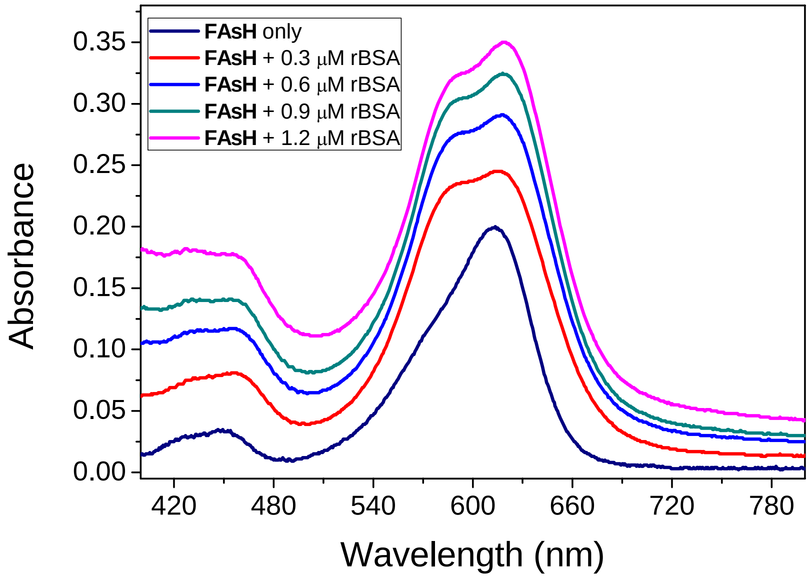

Figure S9. Absorption spectra of **FAsH** (10 μM) in the presence of different concentrations of rBSA (0 – 1.2 μM) in phosphate buffer (20 mM, pH 7.4, containing 1% acetone as cosolvent).

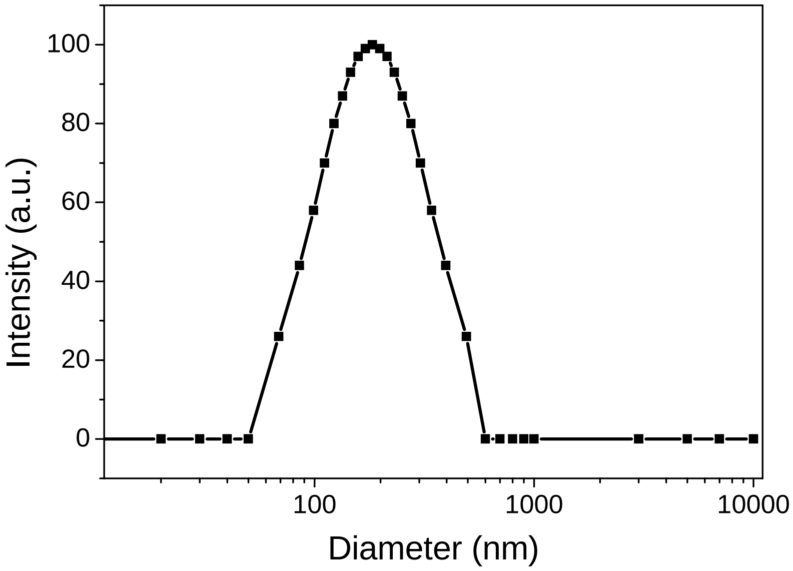

Figure S10. DLS analysis of the particle size distribution of **FAsH** (2.0 μM) in the presence of 1.5 μM rBSA in phosphate buffer (20 mM, pH 7.4). The solution was kept at room temperature (25 ^{o}C) for 20 min prior to taking the measurements.

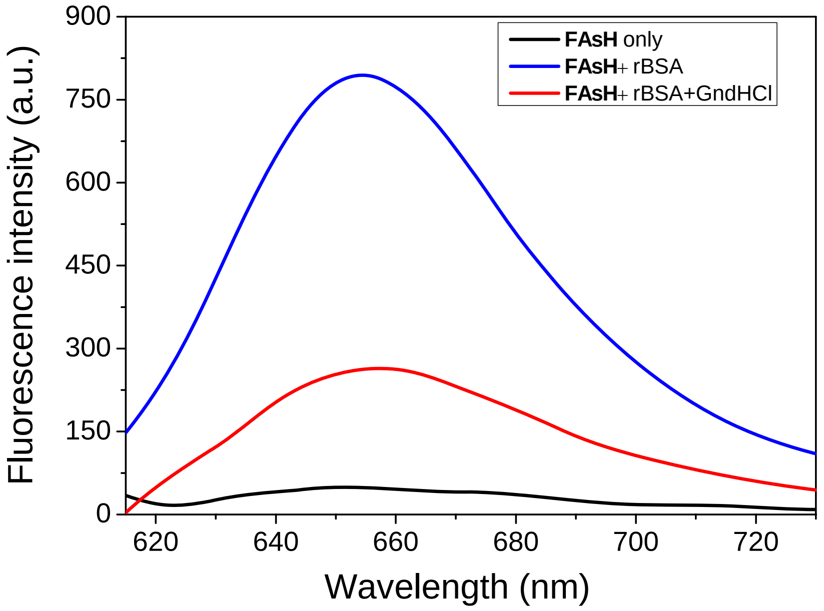

Figure S11. Fluorescence spectra (λ_{ex} = 600 nm) of **FAsH** (1.0 μM) with the addition of rBSA (0.9 μM) and subsequently treating with GndHCl (3.0 M) in phosphate buffer (20 mM, pH 7.4, containing 1% acetone as cosolvent). Each spectrum was recorded 5 min after the addition of the test species.

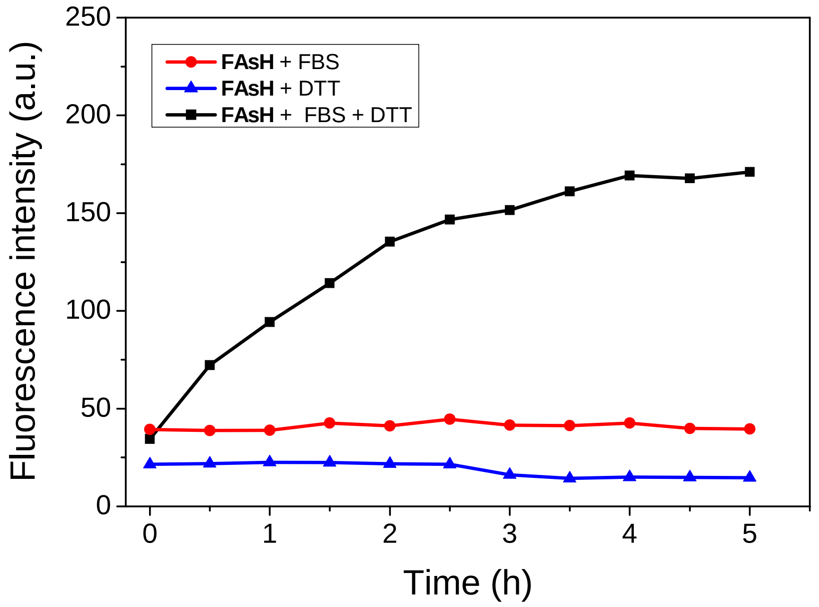

Figure S12. Time course of the fluorescence intensity of **FAsH** (0.5 μM) in the presence of FBS (0.3 %), DTT (5 mM) or the mixture of FBS (0.3 %) and DTT (5 mM) in phosphate buffer (20 mM, pH 7.4, containing 0.5% acetone as cosolvent). λ_{ex} / λ_{em} =621/651 nm.

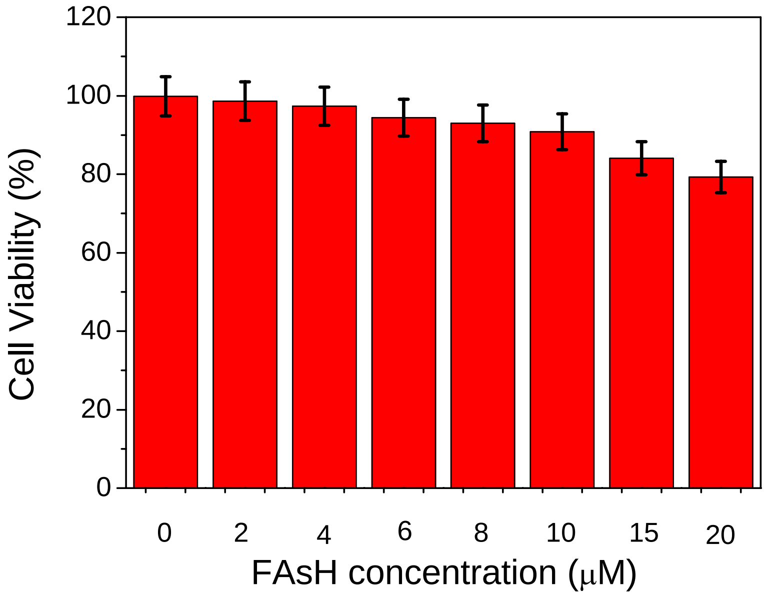

Figure S13. Cell viability estimated by MTT assay. SMMC-7721cells were incubated with different concentrations of **FAsH** (0 - 20 μM) for 12 h.

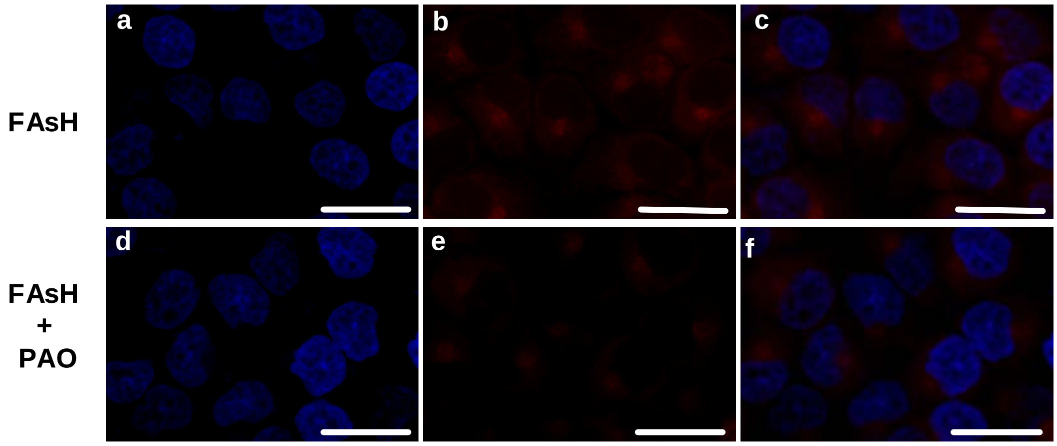

Figure S14. Confocal fluorescence images of intracellular VDPs in SMMC-7721 cells by **FAsH**. (a) and (d), The cells were stained with DAPI (1.0 μg mL^{-1}); (b) the cells were stained with **FAsH** (2.5 μM) for 10 min; (e) the cells were pretreated with **PAO** (30 μM) for 30 min, and then incubated with **FAsH** (2.5 μM) for 10 min; (c), overlay of (a) and (b); (f), overlay of (d) and (e). Scale bar: 15 μm.

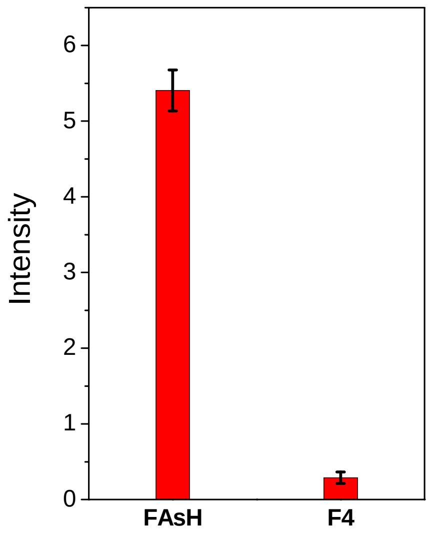

Figure S15. Semiquantitative determination of endogenous VDPs in SMMC-7721 cells according to the averaged fluorescence intensity (650 - 750 nm). The calculations were conducted by ImageJ software. Error bars represent SD.

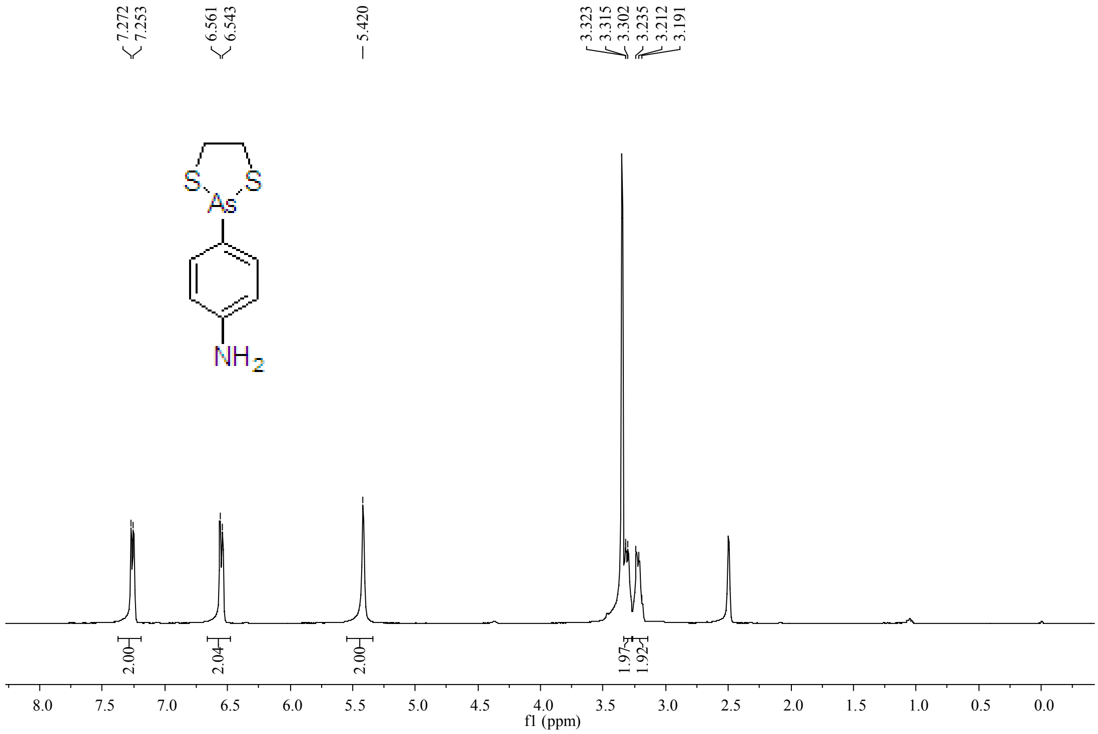

Figure S16. ^{1}H NMR of **PAO-EDT** in DMSO-d_6

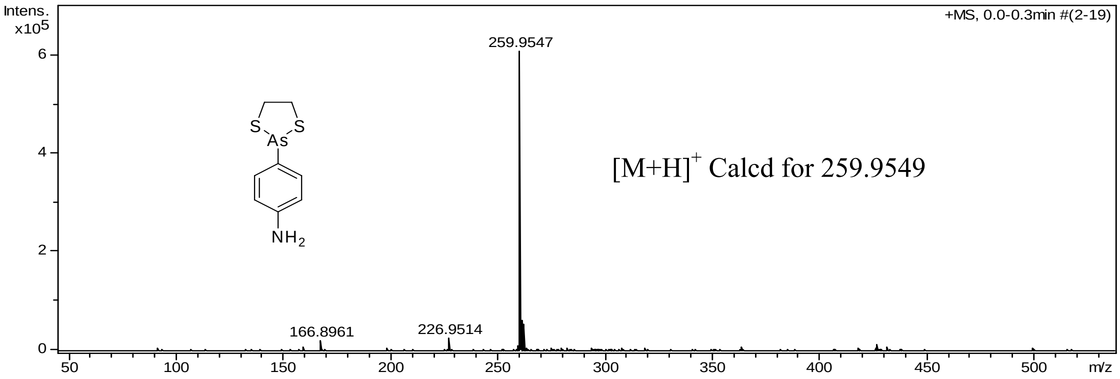

Figure S17. HRMS of **PAO-EDT**

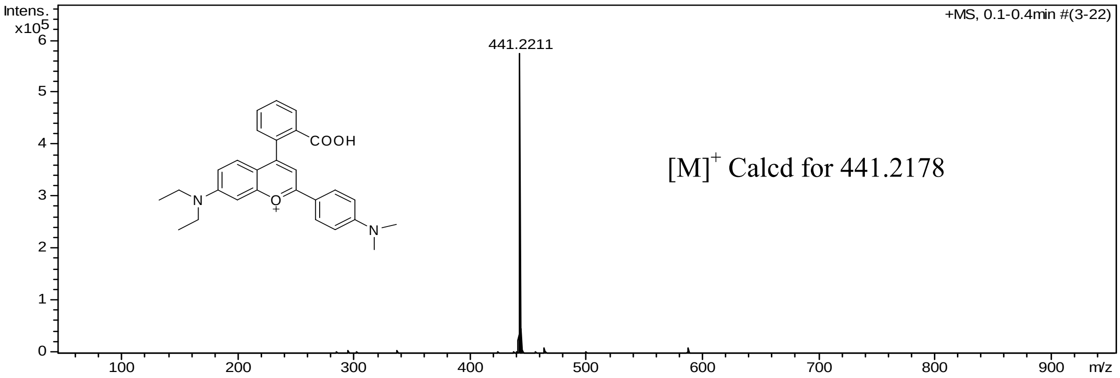

Figure S18. HRMS of **F1**.

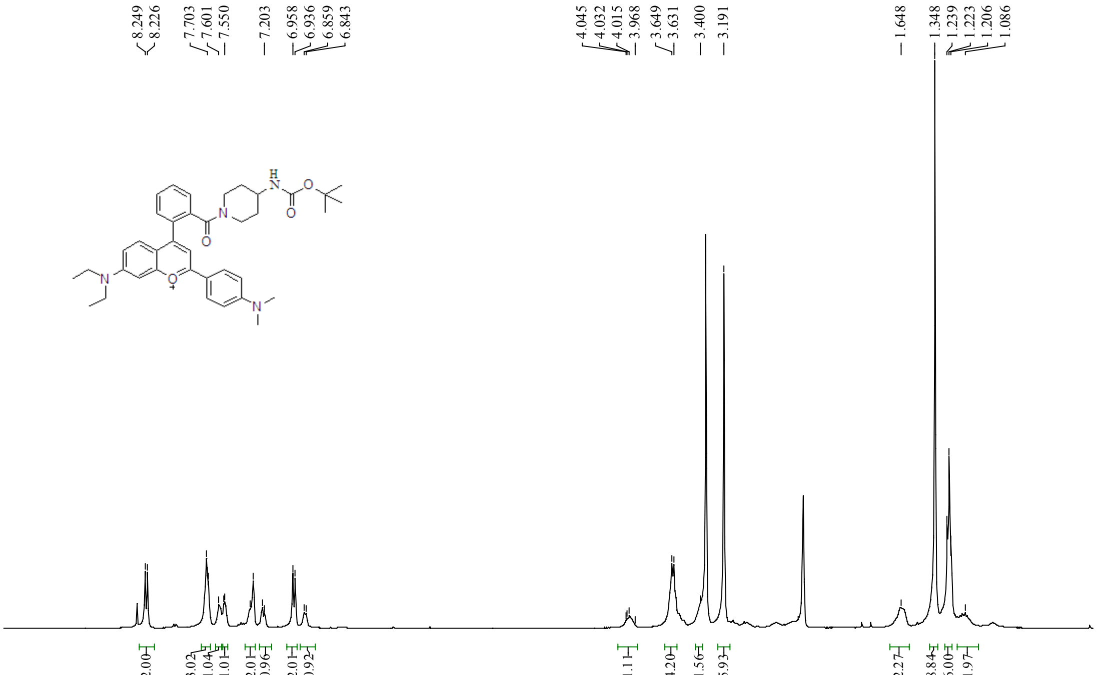

Figure S19. ^{1}H NMR of **F2** in DMSO-d_6.

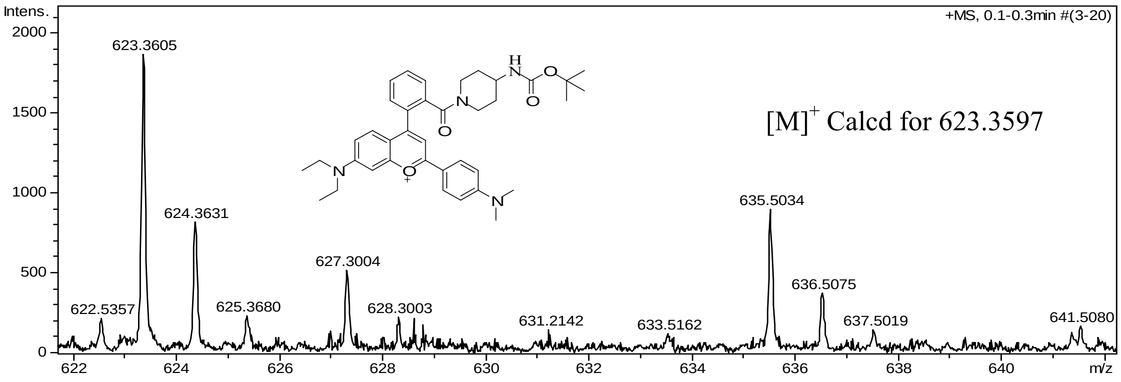

Figure S20. HRMS of **F2.**

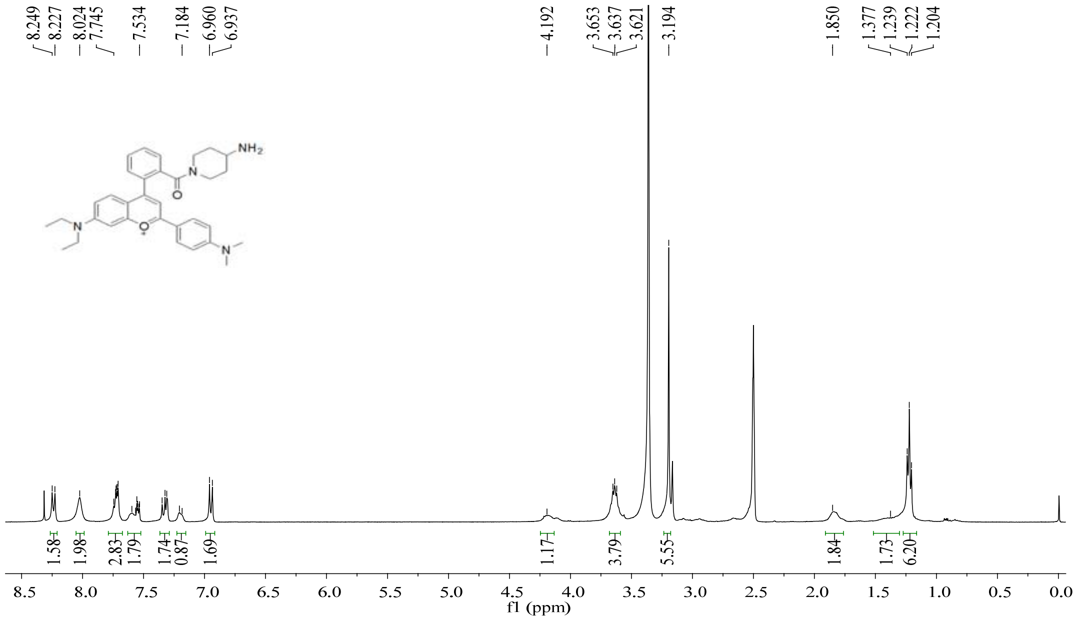

Figure S21. ^{1}H NMR of **F3** in DMSO-d_6.

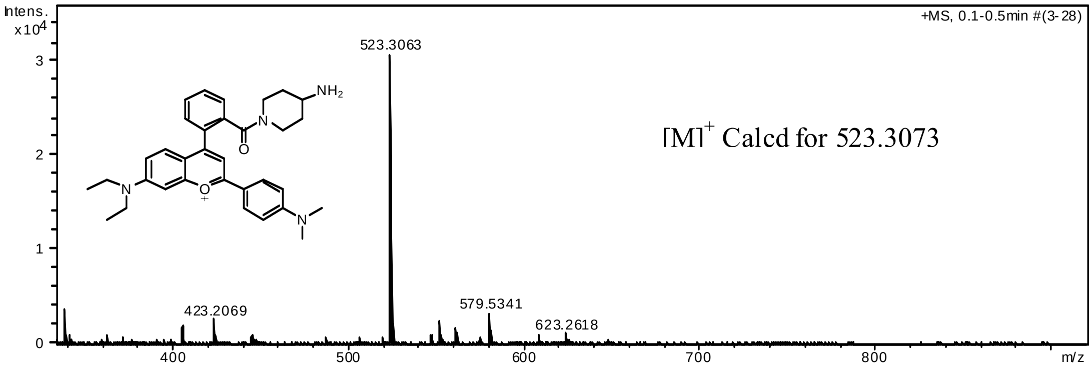

Figure S22. HRMS of **F3**

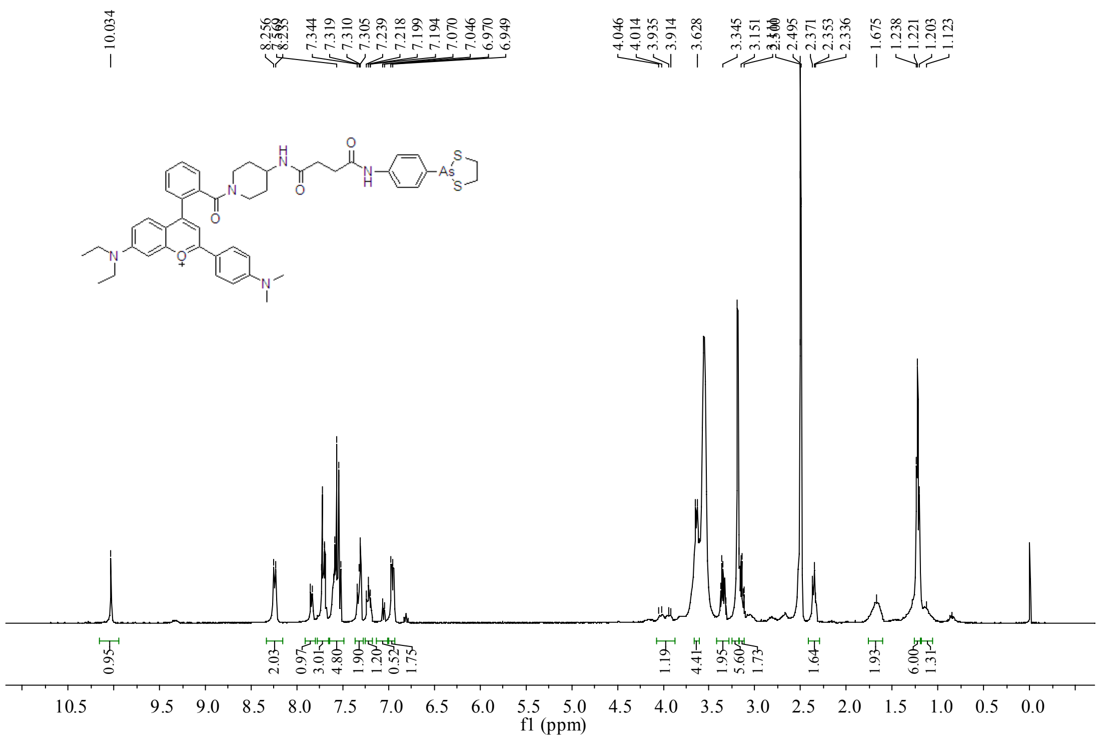

Figure S23. ^{1}H NMR of **FAsH** in DMSO-d_6

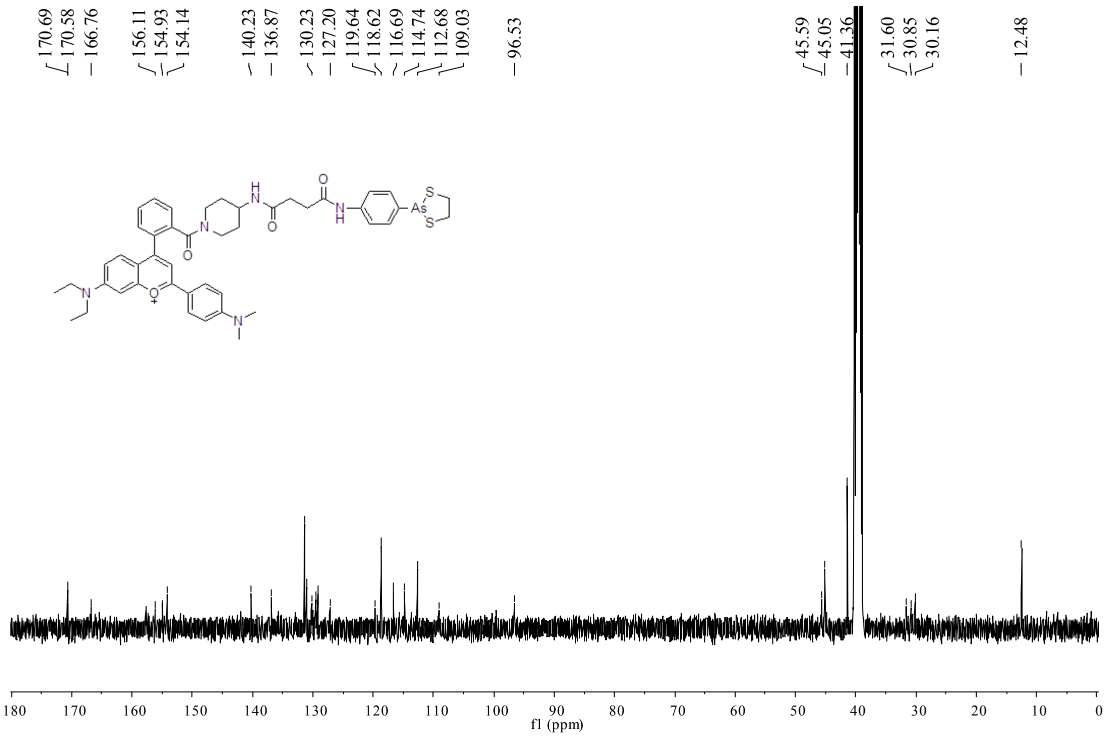

Figure S24. ^{13}C NMR of **FAsH** in DMSO-d_6

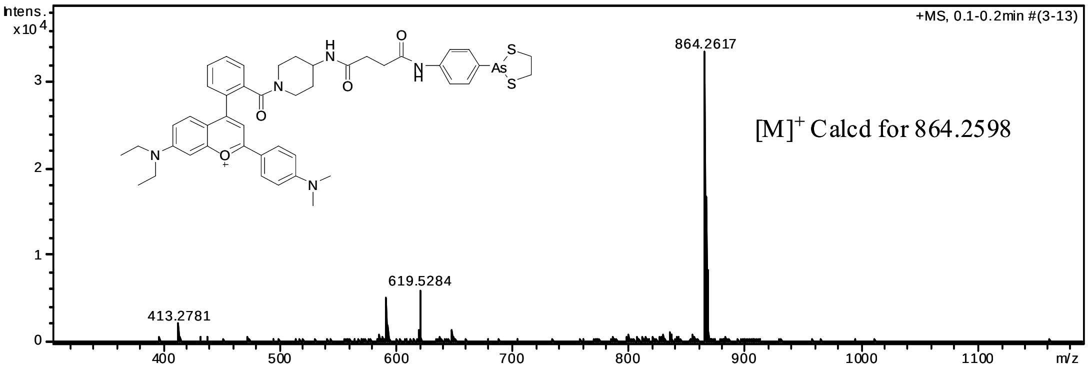

Figure S25. HRMS of **FAsH**

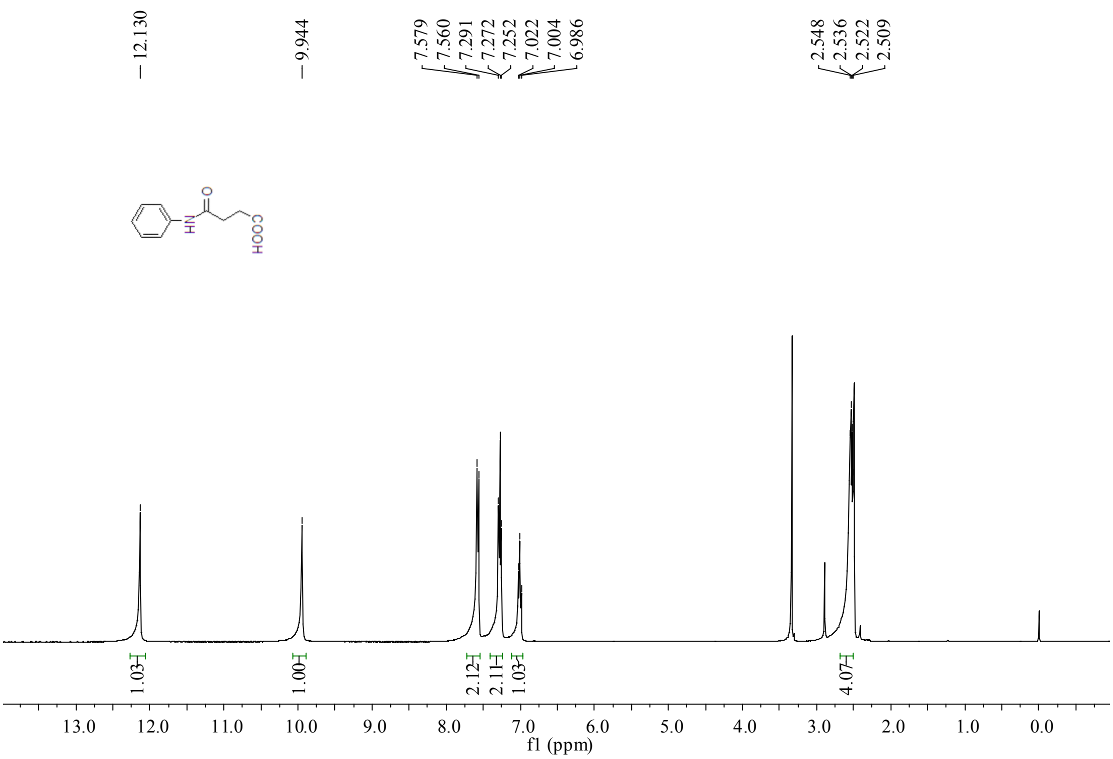

Figure S26. ^{1}H NMR of **2** in DMSO-d_6

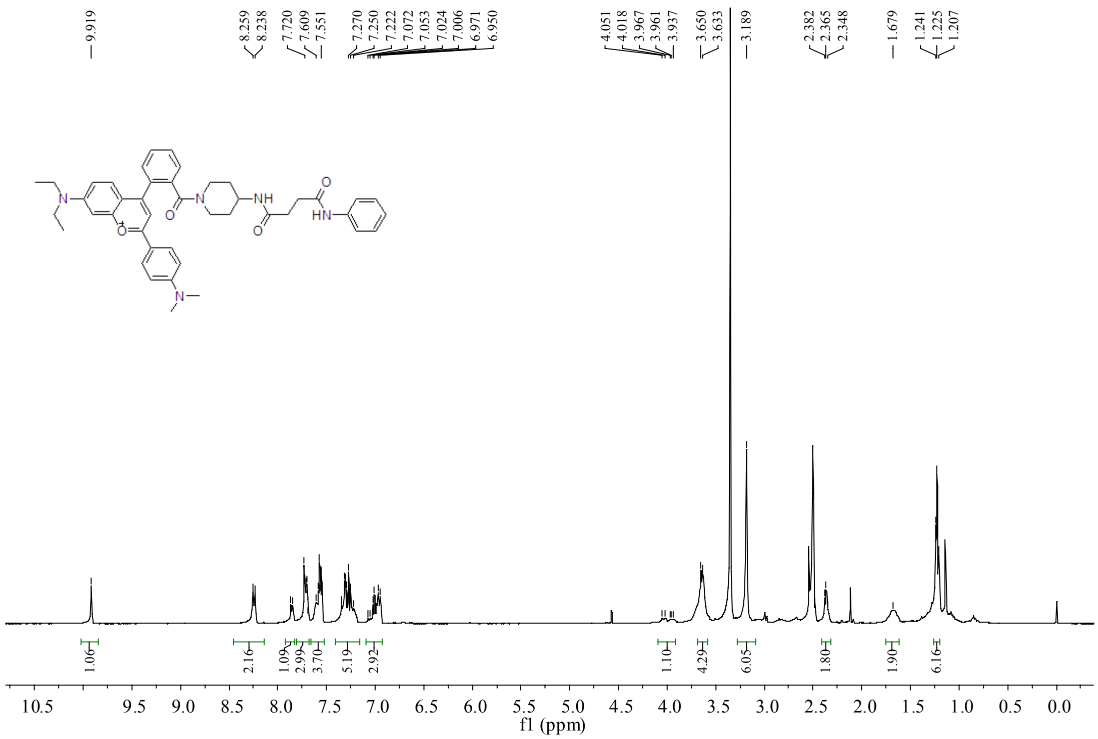

Figure S27. ^{1}H NMR of **F4** in DMSO-d_6

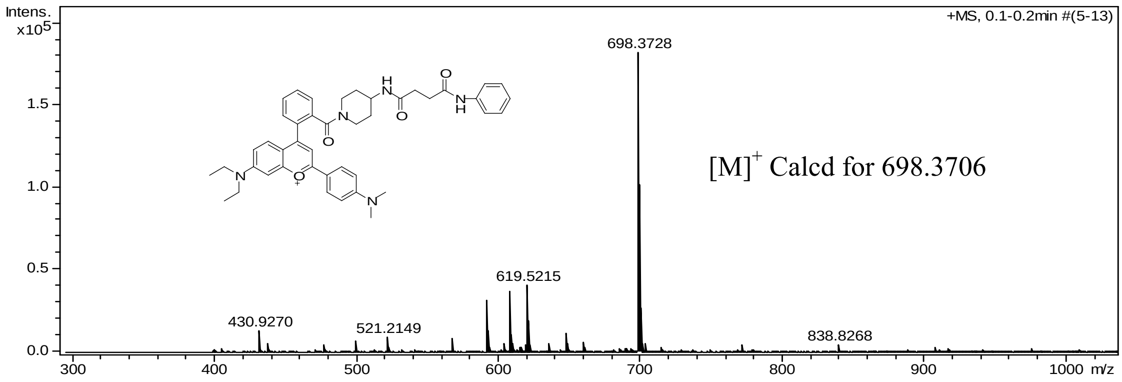

Figure S28. HRMS of **F4**.
